# Supplementary material for: Antigen presentation by lung epithelial cells directs CD4+ TRM cell function and regulates barrier immunity
Source: Nat Commun. 2021 Oct 5;12:5834. doi: 10.1038/s41467-021-26045-w (PMC8492657; doi:10.1038/s41467-021-26045-w)
Supplement: Supplementary file 1 — Supplementary Information [file 41467_2021_26045_MOESM1_ESM.pdf]

SUPPLEMENTARY INFORMATION

Antigen presentation by lung epithelial cells governs $CD4^+$ T_{RM} cell activities and regulates barrier immunity

Anukul T. Shenoy, Carolina Lyon De Ana, Emad I. Arafa, Isabelle Salwig, Kimberly A. Barker, Filiz T. Korkmaz, Aditya Ramanujan, Neelou S. Etesami, Alicia M. Soucy, Ian M.C. Martin, Brian R. Tilton, Anne Hinds, Wesley N. Goltry, Hasmeena Kathuria, Thomas Braun, Matthew R. Jones, Lee J. Quinton, Anna C. Belkina, and Joseph P. Mizgerd.

SUPPLEMENTARY FIGURES

Supplementary Fig. 1

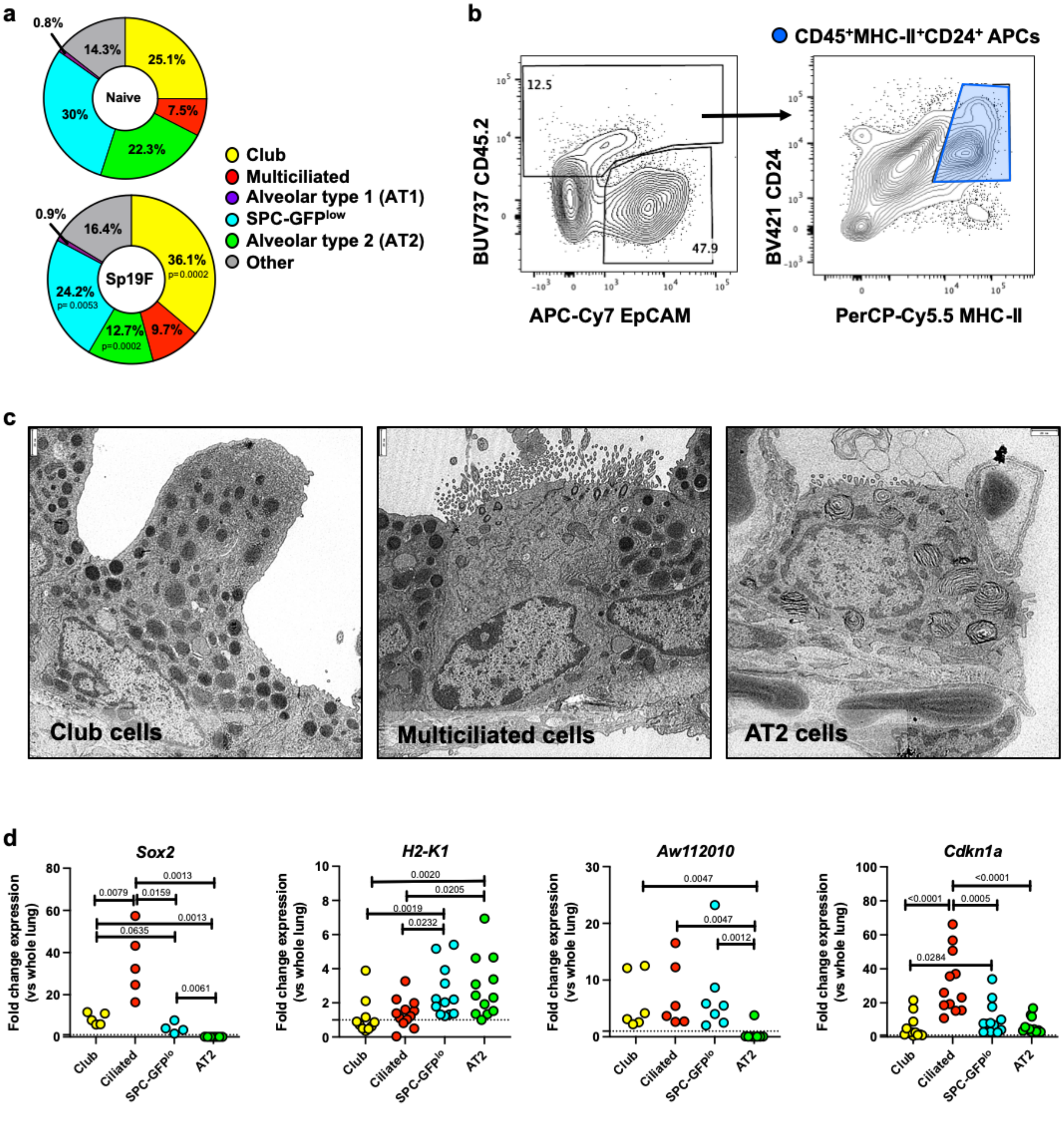

Supplementary Figure. 1: Characterization of distinct LEC subsets. a. Pie charts for mean frequency of LECs recovered from naïve or Sp19F-infected SPC-GFP mouse lungs 48 hours post infection (hpi), two-tailed Mann-Whitney test. **b.** Gating strategy for identification of dendritic cells (DCs) as $CD45^{+}MHC\text{-}II^{+}CD24^{+}$ cells in naïve SPC-GFP lungs. **c.** Representative transmission electron microscopy images of LECs in C57BL/6J mice. >100 anatomically distinct areas of n=6 mouse lungs across independent

experiments were imaged and lamellar bodies were consistently segregated to alveolar LECs and excluded from airways. **d.** mRNA levels of select H2-K1^{high}-club cell related transcripts in sorted LECs, two-tailed Mann-Whitney test. All data have n≥5 mice, 2 independent experiments. Sorting strategy is the same as gating strategy in **Figure. 1a**. All data are presented as mean ± SEM.

Supplementary Fig. 2

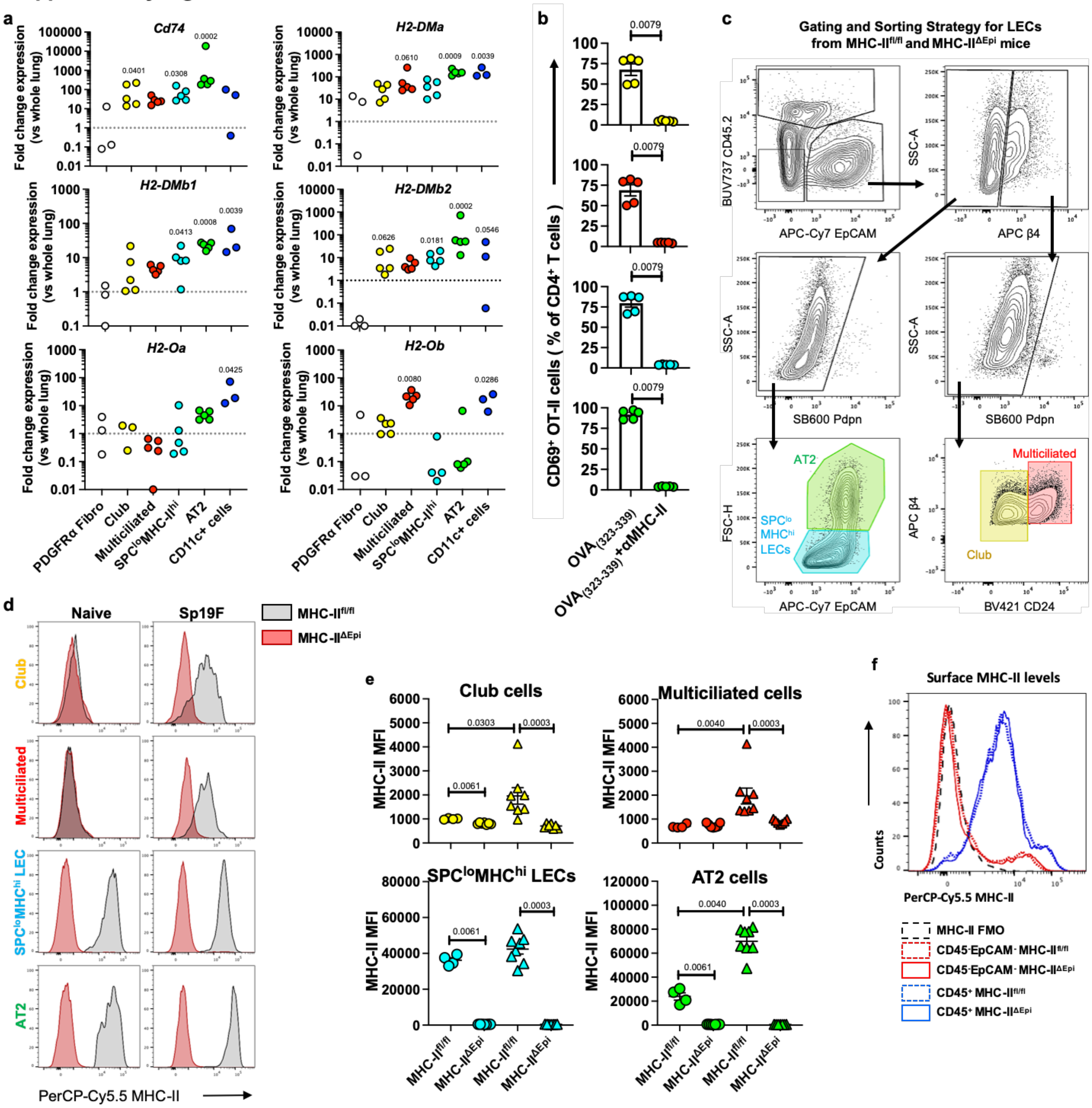

Supplementary Figure. 2: All LECs are bonafide APCs. a. mRNA levels of MHC-II accessory molecules in LECs sorted from SPC-GFP mouse lungs 48 hpi, Kruskal-Wallis test without correction for multiple comparsions. Sorting strategy is the same as gating strategy in **Figure. 1a**. mRNA profiles were compared to CD11c$^+$ cells (positive control for APC activity) and PDGFRα$^+$ fibroblasts (negative control for APC activity) sorted from naïve lungs. Consistent with LECs being bonafide APCs, all LECs expressed CD74 (invariant chain), alpha and beta chains of H2-DM (positive regulator of MHC-II antigen loading pathway) when compared to PDGFRα$^+$ fibroblasts and downregulated expression of alpha and beta chains of H2-DO (negative regulator of MHC-II antigen loading pathway) when compared to CD11c$^+$ cells. p-values on comparison with PDGFRα$^+$

fibroblasts are indicated. **b.** Quantification of OT-II $CD4^+$ T cell activation upon co-culture with $OVA_{323-339}$ and the indicated LECs that had been sorted from Sp19F-infected mice 48hours post infection (hpi), in presence and absence of MHC-II blockade, two-tailed Mann-Whitney test. **c.** Gating and sorting strategy for LECs from MHC-$II^{fl/fl}$ and MHC-$II^{\Delta Epi}$ mice. **d.** Histogram and **e.** Quantification for MHC-II levels on LECs post tamoxifen administration in MHC-$II^{fl/fl}$ and MHC-$II^{\Delta Epi}$ lungs of naïve (circles) and Sp19F infected (48hpi, triangles) mice, two-tailed Mann-Whitney test. **f.** Representative histogram of MHC-II expression on $CD45^+$ and $CD45^-EPCAM^-$ fractions within MHC-$II^{fl/fl}$ and MHC-$II^{\Delta Epi}$ lungs post tamoxifen administration. All data have n≥4 mice, 2 independent experiments. All data are presented as mean ± SEM.

Supplementary Fig. 3

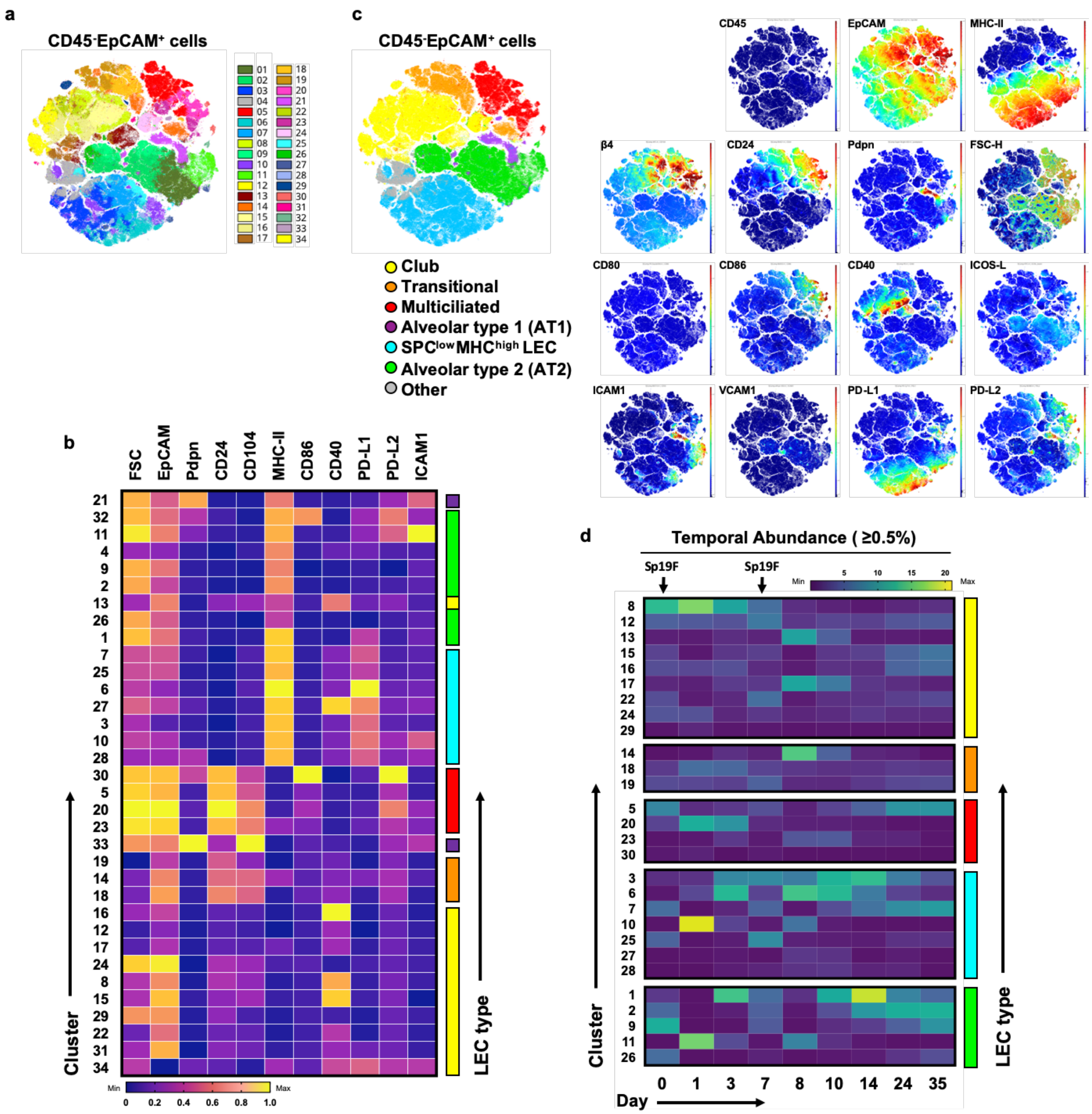

Supplementary Figure. 3: Identification of distinct LEC metaclusters using Phenograph algorithm. a. Phenograph clustering of 34 LEC clusters overlaid on the opt-SNE plot projection of multidimensional cytometry dataset constructed from ~2 million CD45$^-$EpCAM$^+$ LECs isolated from n=84 C57BL6/J mice across 9 timepoints each with 2 indepdendent experiments. **b.** Heatmap depicting normalized expression of surface molecules on 34 LEC clusters (depicted on *left*) spanning 6 metaclusters (depicted on *right*). **c.** opt-SNE plot depicting 6 major LEC metaclusters and expression patterns of all LEC lineage markers and antigen presentation molecules. **d.** Hierarchically clustered heat map depicting mean abundance of distinct LEC clusters within each metacluster

expressed as percent fraction of all LECs at designated time points. LEC clusters are depicted on *left* and the metaclusters are depicted on *right*.

Supplementary Fig. 4

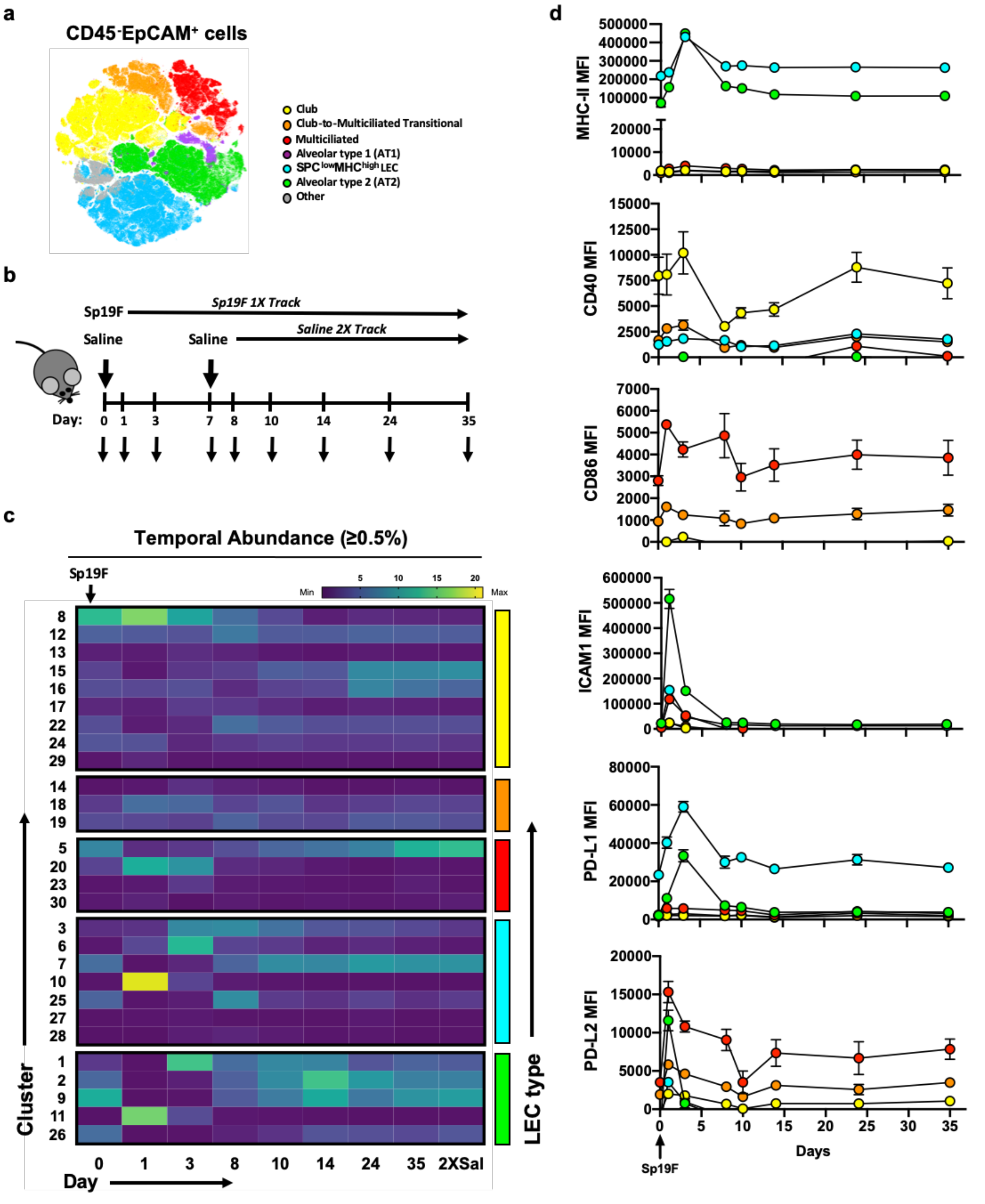

Supplementary Figure. 4: Single *Spn* infection does not incite changes in cell surface levels of APC molecules on LECs on day 8. a. opt-SNE plot depicting 6 major LEC metaclusters. **b.** Schematic of timeline for the controls pertaining to the study

described in **Fig. 2**. Controls were divided into 2 tracks: one track that received 1X Sp19F infection and analyzed at designated timepoints and another track that received 2X Saline exposures and analyzed at day 35 (2X Sal). **c.** Heat map depicting mean abundance of distinct LEC clusters within each metacluster expressed as percent fraction of all LECs at designated time points. LEC clusters are depicted on *left* and the metaclusters are depicted on *right*. **d.** Temporal quantification of MHC-II, CD40, CD86, ICAM1, PD-L1, and PD-L2 levels across distinct LECs in the SP19F 1X track. All experiments have n≥5 mice/timepoint, 2 independent experiments. All data are presented as mean ± SEM.

Supplementary Fig. 5

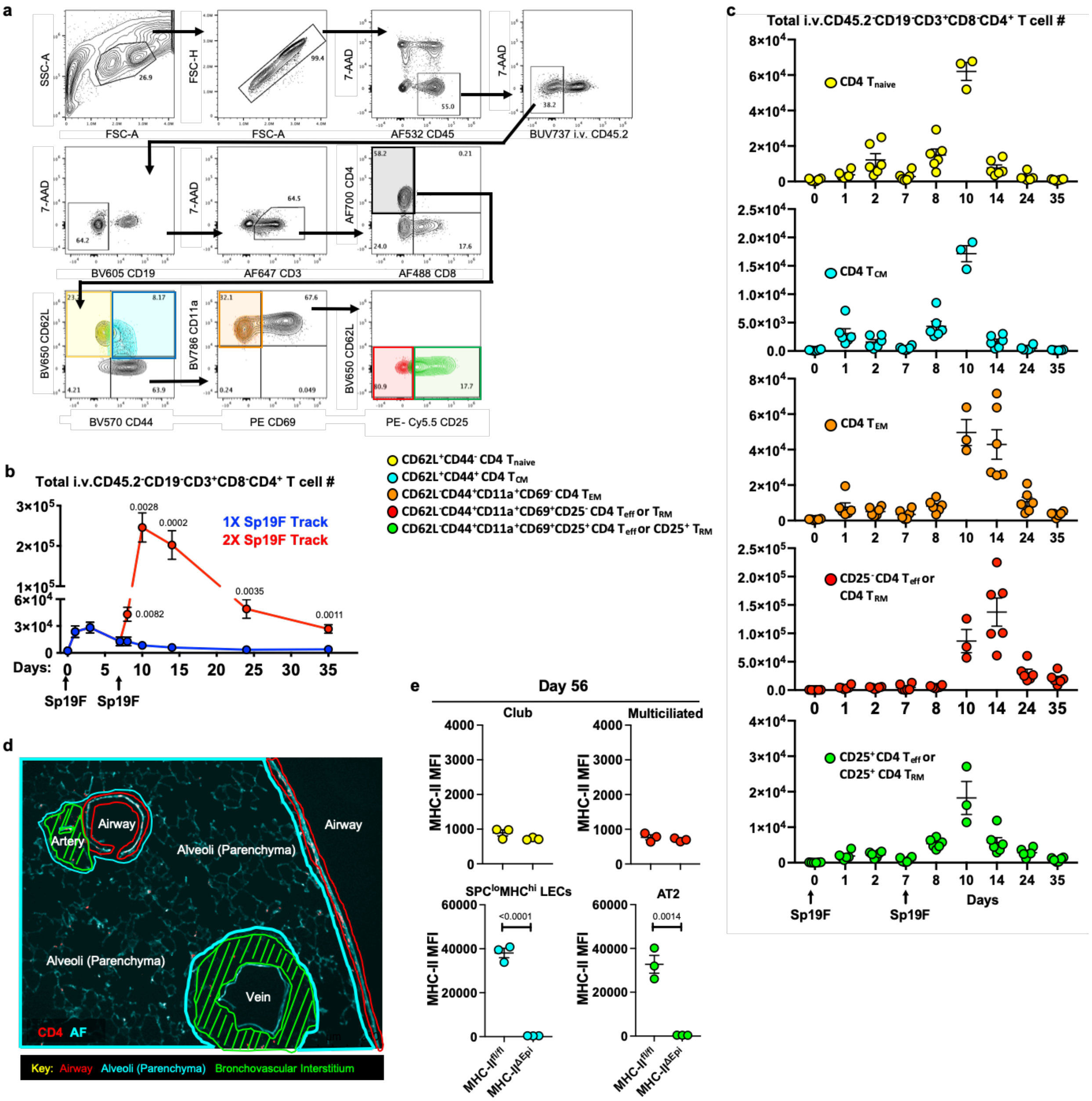

Supplementary Figure. 5: LECs regulate CD4⁺ T_{RM} niche locations. a. Gating strategy for identification of lung (i.v.CD45.2⁻) CD4⁺ T cell subsets. **b.** Absolute numbers of lung (i.v.CD45.2⁻) CD4⁺ T cells at designated timepoints on 1X Sp19F and 2X Sp19F infection tracks. Multiple *t* test with two-stage step-up method of Benjamini, Krieger and Yekutieli to correct for multiple comparisons, n≥5 mice/timepoint, 2 independent experiments, except day 10 with n=3 mice, *p*-value for comparisons with 1X Sp19F track denoted; mean ± SEM. **c.** Absolute numbers of lung (i.v.CD45.2⁻) CD4⁺ T cell subsets described in **Figure. 3a** at designated timepoints on 2X Sp19F infection track. n≥5 mice/timepoint, 2 independent experiments, except day 10 with n=3 mice; mean ± SEM. **d.** A sample immunofluorescent micrograph showing how distinct anatomical structures of lungs were

identified to quantify localization of $CD4^+$ niches at designated timepoints. Area bordered by *green* was identified as bronchovascular interstitium. Area bordered by *red* was identified as airway and area bordered by *cyan* was identified as alveoli (or parenchyma). **e.** MHC-II levels on LEC from experienced MHC-$II^{fl/fl}$ and MHC-$II^{\Delta Epi}$ lungs on day 56. Two-tailed Unpaired *t* test, n=3 mice,1 experiment. All data are presented as mean ± SEM.

Supplementary Fig. 6

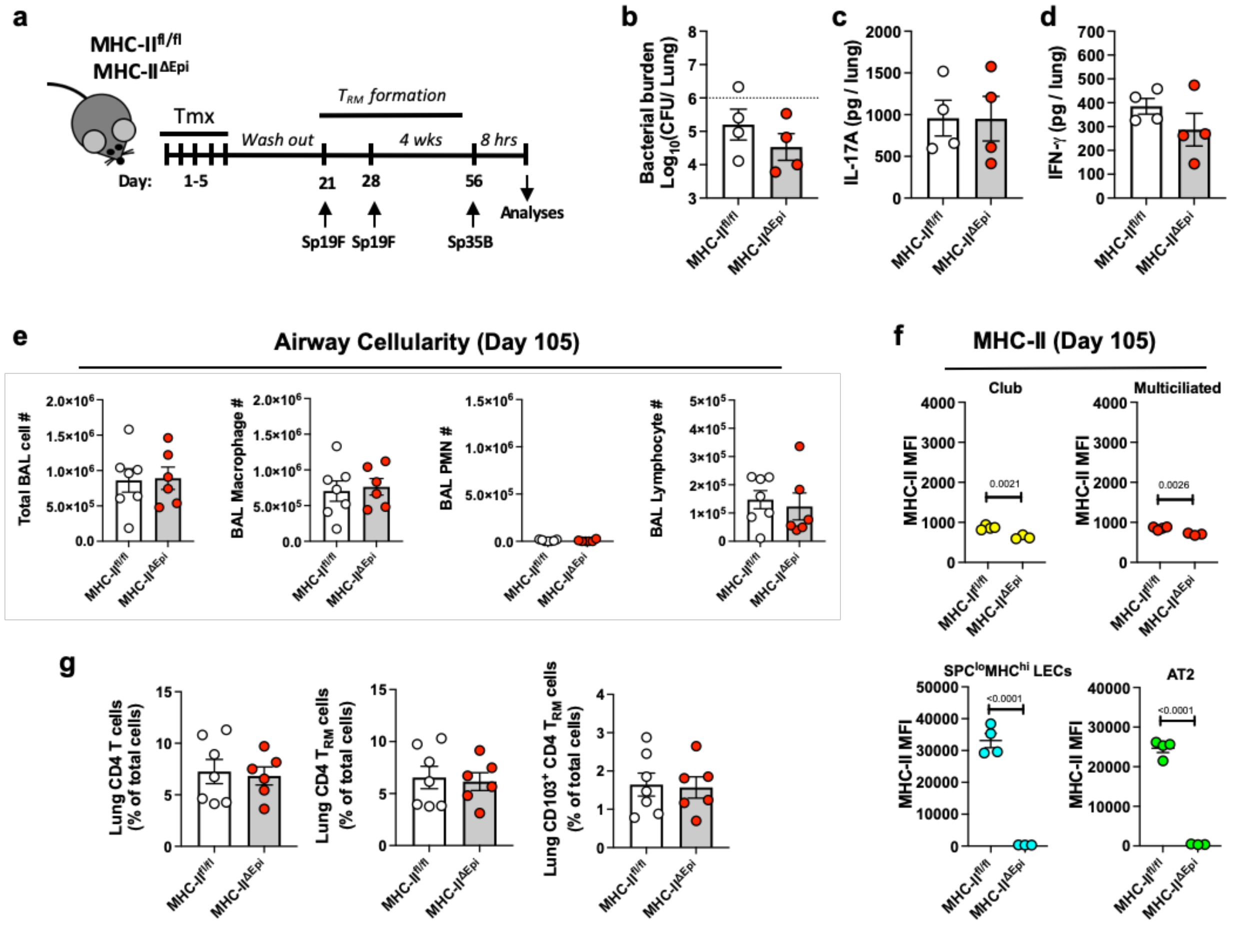

Supplementary Figure. 6: Experienced MHC-II$^{fl/fl}$ and MHC-II$^{\Delta Epi}$ lungs are adept at memory recall and show no signs of overt inflammation on day 105. a. Schematic of experimental timeline used. Quantification of **b.** Whole lung bacterial burden, **c.** IL-17A levels, and **d.** IFN-γ levels 8hpi with Sp35B, n=4 mice, 2 independent experiments. **e.** Total bronchoalveolar lavage (BAL) cellularity, macrophages, neutrophils (PMNs) and lymphocytes on day 105, two-tailed Mann-Whitney test, n≥5 mice, 2 independent experiments. **f.** MHC-II levels on LECs from experienced MHC-II$^{fl/fl}$ and MHC-II$^{\Delta Epi}$ lungs on day 105, two-tailed Unpaired *t* test, n≥3 mice, 1 experiment. **g.** Frequencies of lung (i.v.CD45.2^{-}) CD4^{+} T cells, CD69^{+}CD11a^{high} CD4^{+} T_{RM} cells and CD103^{+} CD4^{+} T_{RM} cells in lungs of experienced MHC-II$^{fl/fl}$ and MHC-II$^{\Delta Epi}$ mice at day 105, two-tailed Mann-Whitney test, n≥5 mice, 2 independent experiments. All data are presented as mean ± SEM.

Supplementary Fig. 7

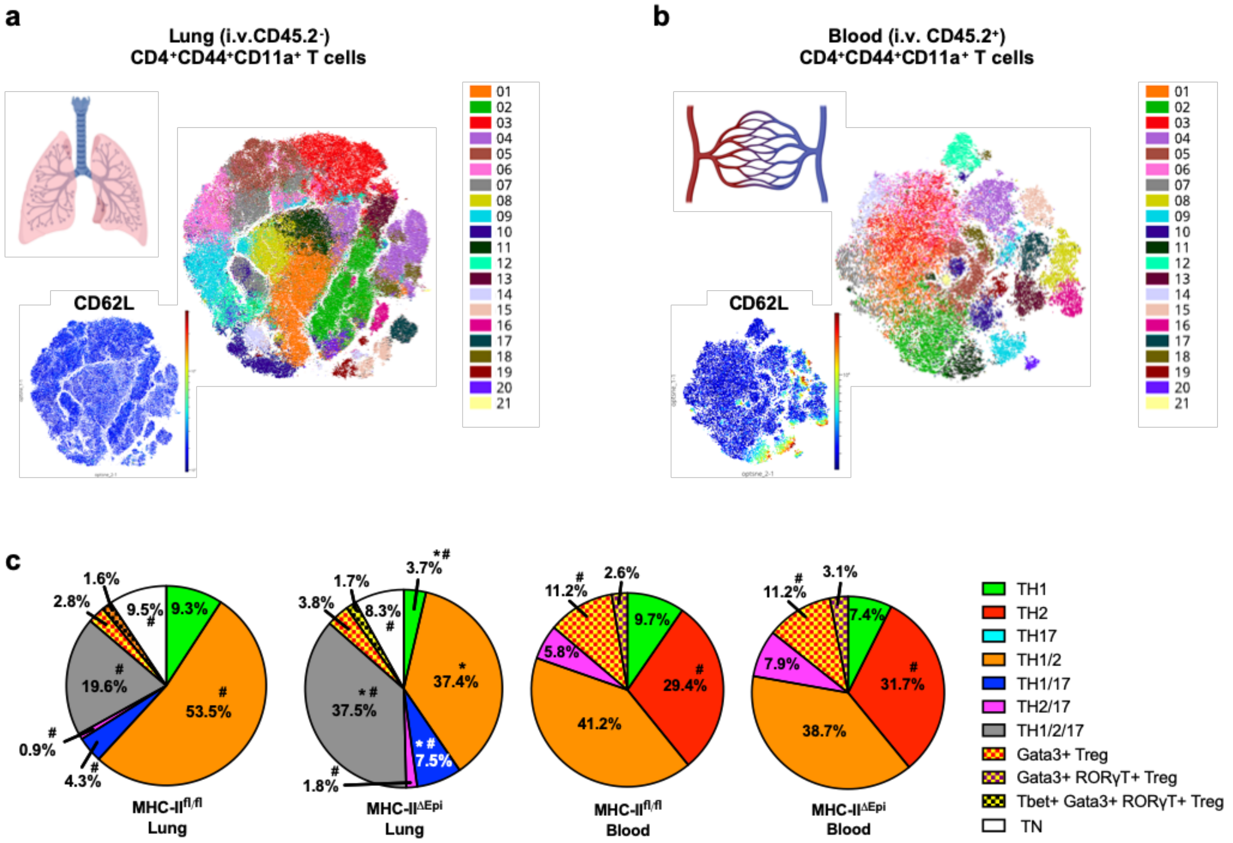

Supplementary Figure. 7: LEC MHC-II constrains aberrant expansion of multipotent CD4^{+} T$_{RM}$ cells. a. Phenograph clustering overlaid on opt-SNE projection depicting lung (i.v.CD45.2^{-}) memory (CD44^{+}CD11a^{+}) CD4^{+} T cells concatenated from MHC-II$^{fl/fl}$ and MHC-II$^{\Delta Epi}$ lungs on day 105. ***Inset:*** opt-SNE depicting CD62L expression on lung memory CD4^{+} T cells. **b.** Phenograph clustering overlaid on opt-SNE projection depicting intravascular (i.v.CD45.2^{+}) memory (CD44^{+}CD11a^{+}) CD4^{+} T cells concatenated from MHC-II$^{fl/fl}$ and MHC-II$^{\Delta Epi}$ lungs on day 105. ***Inset:*** opt-SNE depicting CD62L expression and identifying T$_{CM}$ populations within lung memory CD4^{+} T cells. **c.** Pie charts for mean frequencies of distinct memory T$_{H}$ cell lineages within lungs and blood of MHC-II$^{fl/fl}$ and MHC-II$^{\Delta Epi}$ mice at day 105, Two-way ANOVA with Fisher's LSD test. *p*-value: $^{\#}\leq 0.05$ for comparison between lung and blood; $^{*}\leq 0.05$ for genotype-dependent comparison within lungs; $^{\Phi}\leq 0.05$ for genotype-dependent comparison within blood. Positivity for a LDTF was determined using cutoffs identified from clusters negative for that LDTF. All data have n≥6 mice, 2 independent experiments. Lung and blood schematic were created with BioRender.com.

Supplementary Fig. 8

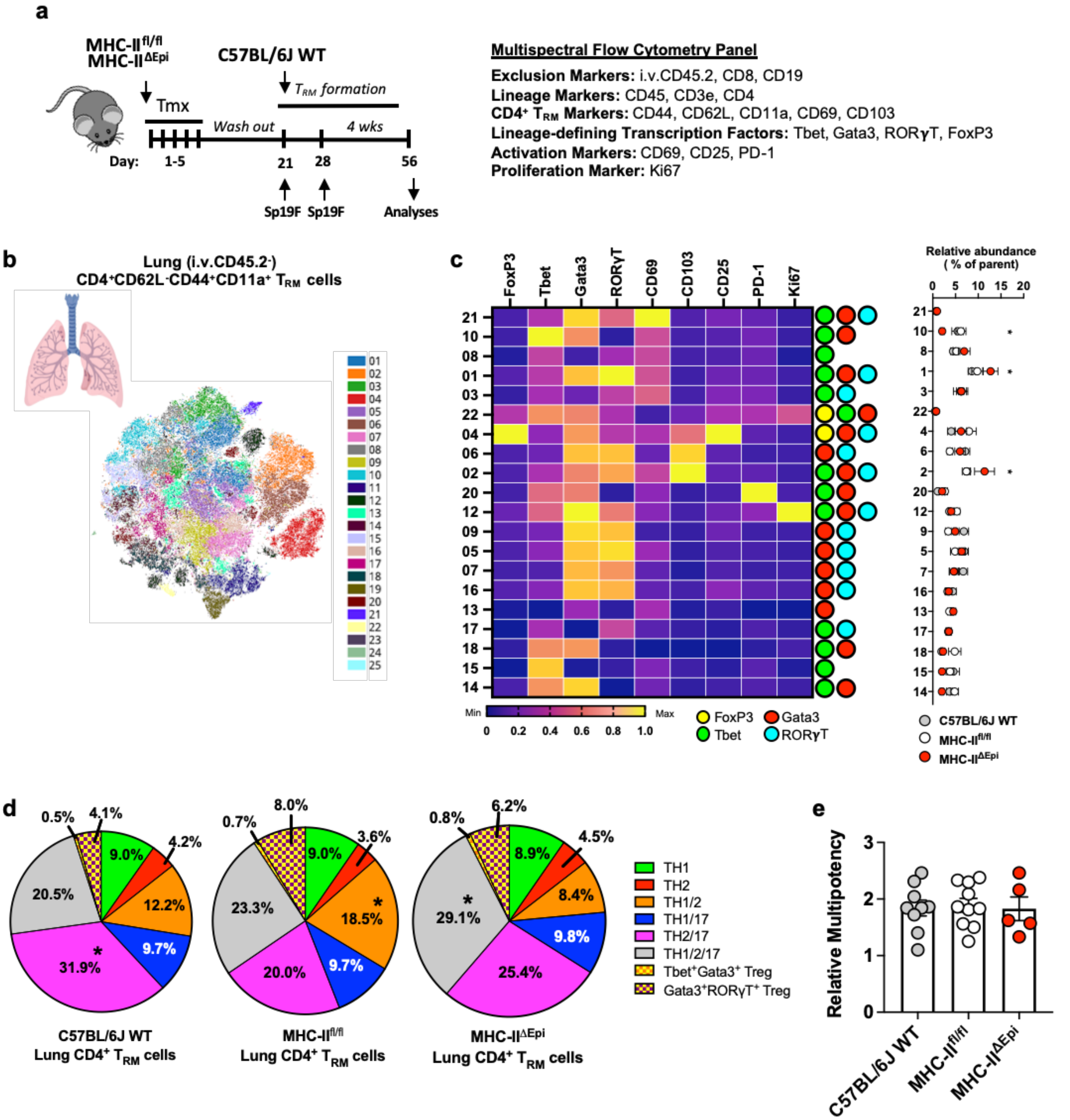

Supplementary Figure. 8: CD4$^+$ T$_{RM}$ cells are plastic since their establishment. a. Schematic of experimental timeline. **b.** Phenograph clustering overlaid on opt-SNE projection depicting lung (i.v.CD45.2$^-$) CD6L$^-$CD44$^+$CD11a$^+$ CD4$^+$ T$_{RM}$ cells concatenated from experienced C57BL/6J WT, MHC-II$^{fl/fl}$ and MHC-II$^{\Delta Epi}$ lungs on day 56. **c.** ***Left:*** Heat map depicting normalized expression levels of distinct molecules on lung (i.v.CD45.2$^-$) memory CD4$^+$ T$_{RM}$ cells clusters on day 56. Lineage determining transcription factor (LDTF) status of each cluster is depicted. ***Right:*** Relative abundance of each cluster at day 56 in C57BL/6J WT, MHC-II$^{fl/fl}$ and MHC-II$^{\Delta Epi}$ lungs. Two-way ANOVA with two-stage step-up method of Benjamini, Krieger and Yekutieli to correct for multiple comparisons.

FDR *p* value: * ≤ 0.05. **d.** Pie charts for mean frequencies of distinct T_{RM} cell lineages within lungs of C57BL/6J WT, MHC-$II^{fl/fl}$ and MHC-$II^{\Delta Epi}$ mice at day 56, Two-way ANOVA with Fisher's LSD test. *p*-value: *≤ 0.05 for genotype-dependent comparison within lungs. **e.** Relative multipotency indices for $CD4^+$ T_{RM} cells in lung of C57BL/6J WT (*gray*), MHC-$II^{fl/fl}$ (*white*) and MHC-$II^{\Delta Epi}$ mice (*red*) at day 56. Positivity for a LDTF was determined using cutoffs identified from clusters negative for that LDTF. All data have n≥5 mice, 2 independent experiments. All data are presented as mean ± SEM. Lung schematic created with BioRender.com.

Supplementary Fig. 9

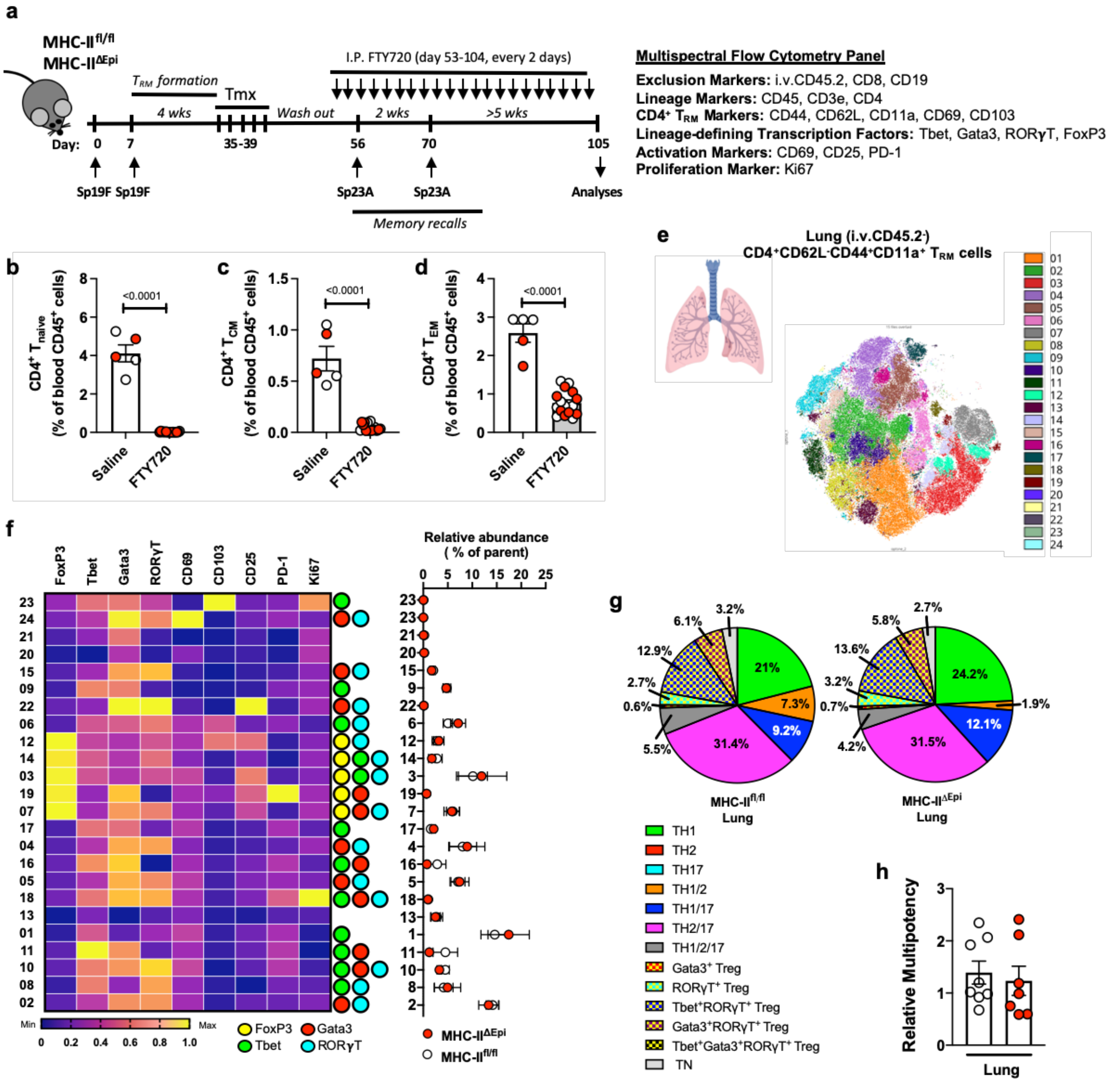

Supplementary Figure. 9: LEC MHC-II regulates the development of multipotent CD4⁺ T_{RM} cells from recruited Teff cells. a. Schematic of experimental timeline. **b.** Quantification of **b.** CD4⁺ T_{Naive}, **c.** CD4⁺ T_{CM} and **d.** CD4⁺ T_{EM} cells in saline and FTY720 treated MHC-II$^{fl/fl}$ and MHC-II$^{\Delta Epi}$ lungs on Day 105. Subsets were identified as in **Supplementary Fig. 5a,** two-tailed Mann-Whitney test. **e.** Phenograph clustering overlaid on opt-SNE projection depicting lung (i.v.CD45.2$^-$) CD6L$^-$CD44$^+$CD11a$^+$ CD4$^+$ T_{RM} cells concatenated from FTY720 treated, experienced MHC-II$^{fl/fl}$ and MHC-II$^{\Delta Epi}$ mice on day 105. **f.** ***Left:*** Heat map depicting normalized expression levels of distinct molecules on lung (i.v.CD45.2$^-$) memory CD4$^+$ T_{RM} cells clusters on day 105. Lineage determining transcription factor (LDTF) status of each cluster is depicted. ***Right:*** Relative abundance of each CD4$^+$ T_{RM} cell cluster from FTY720 treated, experienced MHC-II$^{fl/fl}$ and MHC-II$^{\Delta Epi}$ mice at day 105. No genotype-dependent differences observed. **g.** Pie charts for mean

frequencies of distinct T_{RM} cell lineages within lungs of FTY720 treated, experienced MHC-$II^{fl/fl}$ and MHC-$II^{\Delta Epi}$ mice at day 105, Two-way ANOVA. No genotype-dependent differences observed. **h.** Relative multipotency indices for $CD4^+$ T_{RM} cells in lung of FTY720 treated, experienced MHC-$II^{fl/fl}$ (*white*) and MHC-$II^{\Delta Epi}$ mice (*red*) at day 105. Positivity for a LDTF was determined using cutoffs identified from clusters negative for that LDTF. All data have n≥6 mice, 2 independent experiments. All data are presented as mean ± SEM. Lung schematic created with BioRender.com.

Supplementary Fig. 10

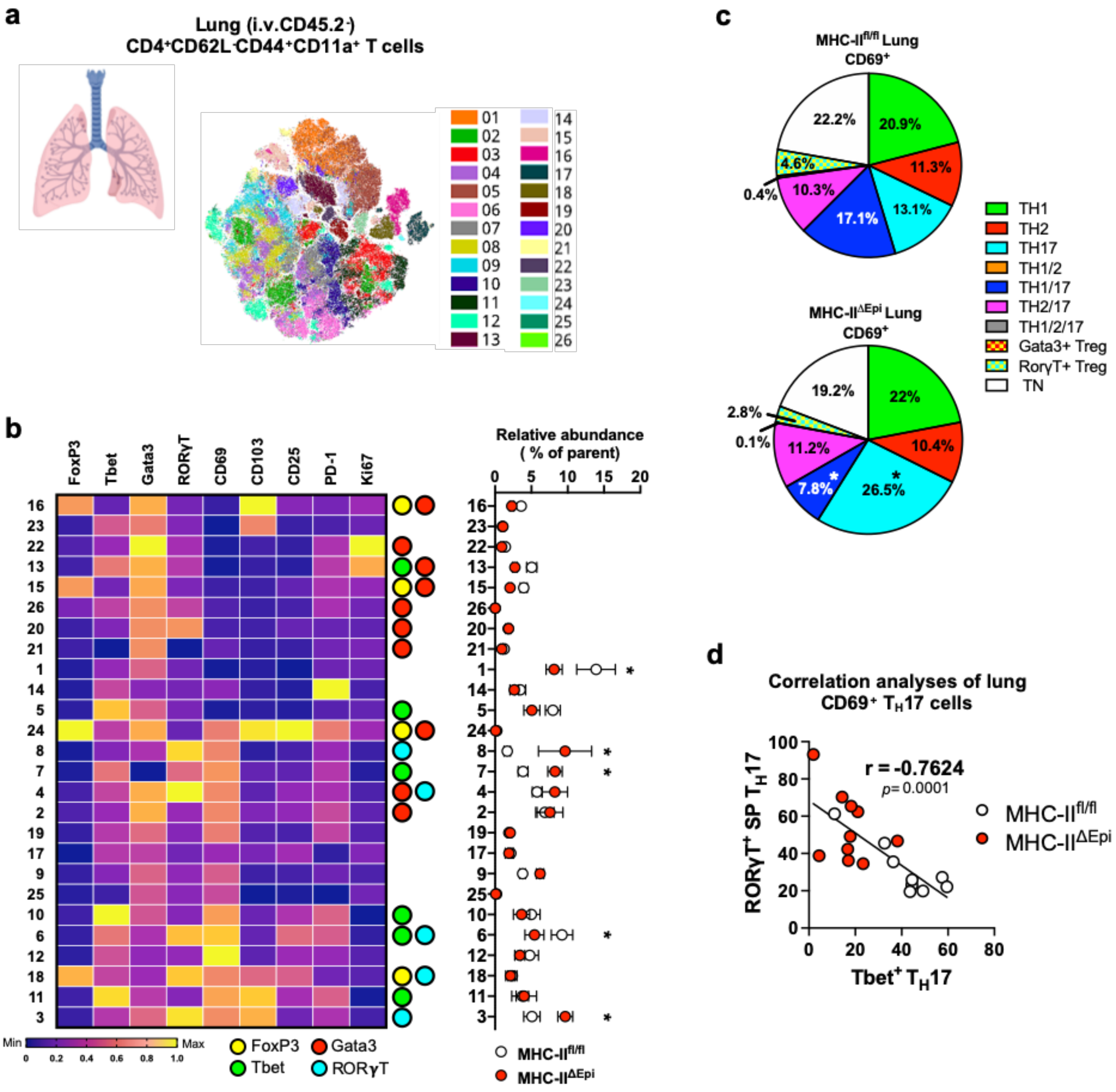

Supplementary Figure. 10: CD4⁺ T_{RM} cells from MHC-II$^{\Delta Epi}$ mice deviate away from T_H1 responses on memory recall. a. Phenograph clustering overlaid on opt-SNE plot depicting lung (i.v.CD45.2$^-$) CD62L$^-$CD44$^+$CD11a$^+$ CD4$^+$ T cells concatenated from MHC-II$^{fl/fl}$ and MHC-II$^{\Delta Epi}$ lungs 8hpi Sp35B. **b. *Left:*** Heat map depicting normalized expression levels of distinct molecules on lung CD4$^+$ T cell clusters. LDTF status of each cluster are depicted. Positivity for a LDTF was determined using cutoffs identified from clusters negative for that LDTF. ***Right:*** Relative abundance of each cluster 8hpi Sp35B. Two-way ANOVA with two-stage step-up method of Benjamini, Krieger and Yekutieli to correct for multiple comparisons. FDR p value: *≤ 0.05. **c.** Pie charts for mean frequencies of distinct CD69$^+$ T_H cell lineages isolated from lungs 8hpi Sp35B, Two-Way ANOVA with Fisher's

LSD test. *p* value: *≤ 0.05 for comparison between genotypes. **d.** Two-tailed Pearson correlation analyses of activated ($CD69^+$) $Tbet^+$ T_H17 cells and RORγT SP T_H17 cells in lungs 8hpi with Sp35B. Positivity for a LDTF was determined using cutoffs identified from clusters negative for that LDTF. All data have n≥8 mice, 2 independent experiments. All data are presented as mean ± SEM. Lung schematic created with BioRender.com.

Supplementary Fig. 11

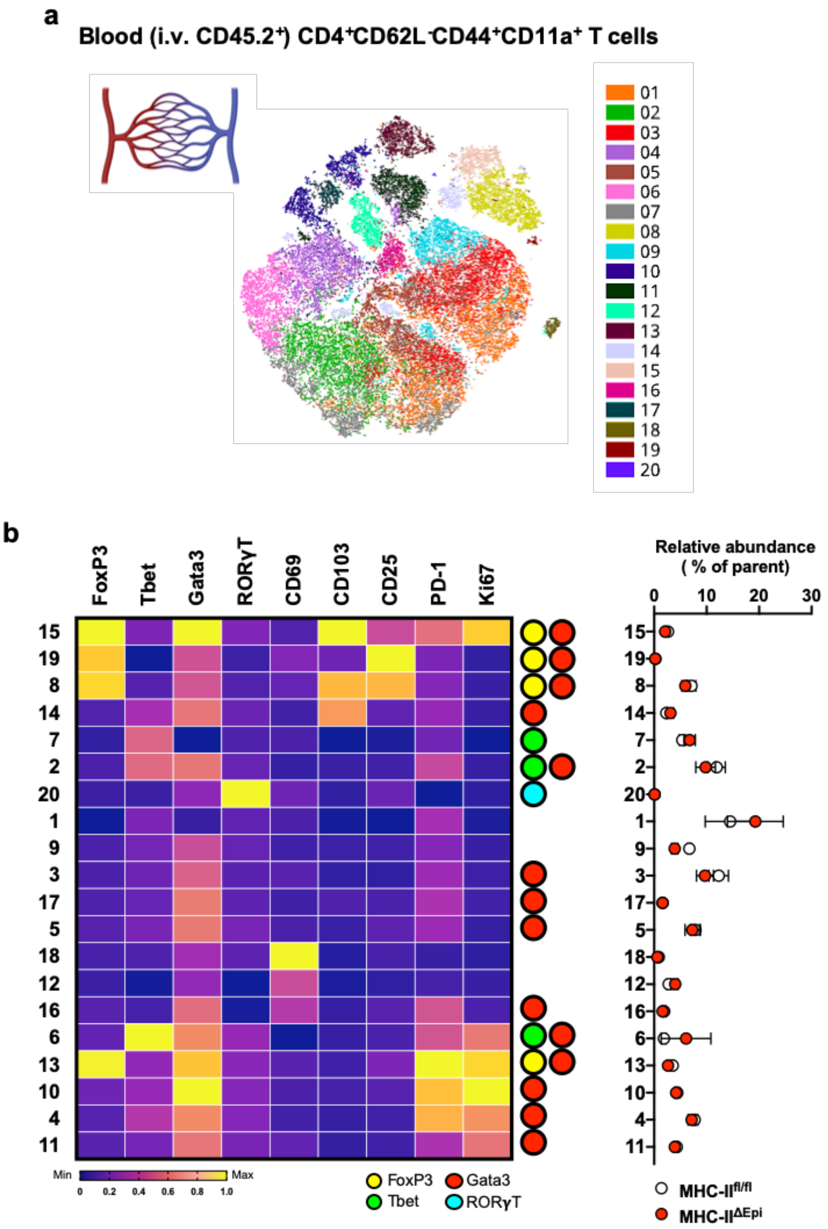

Supplementary Figure. 11: Intravascular CD4$^+$ T cells of experienced MHC-II$^{fl/fl}$ and MHC-II$^{\Delta Epi}$ mice 8hpi with Sp35B. a. Phenograph clustering overlaid on opt-SNE projection depicting intravascular (i.v.CD45.2$^+$) CD62L$^-$CD44$^+$CD11a$^+$ CD4$^+$ T cells concatenated from MHC-II$^{fl/fl}$ and MHC-II$^{\Delta Epi}$ lungs 8hpi Sp35B. **b.** ***Left:*** Heat map depicting normalized expression levels of distinct molecules on identified blood CD4$^+$ T cell clusters 8hpi Sp35B. LDTF status of each cluster is depicted. ***Right:*** Relative abundance of each cluster in blood of experienced MHC-II$^{fl/fl}$ and MHC-II$^{\Delta Epi}$ mice 8hpi Sp35B. No genotype-dependent differences observed.. Positivity for a LDTF was determined using cutoffs identified from clusters negative for that LDTF. All experiments

have n≥6 mice, 2 independent experiments. All data are presented as mean ± SEM. Blood schematic created with BioRender.com.

Supplementary Fig. 12

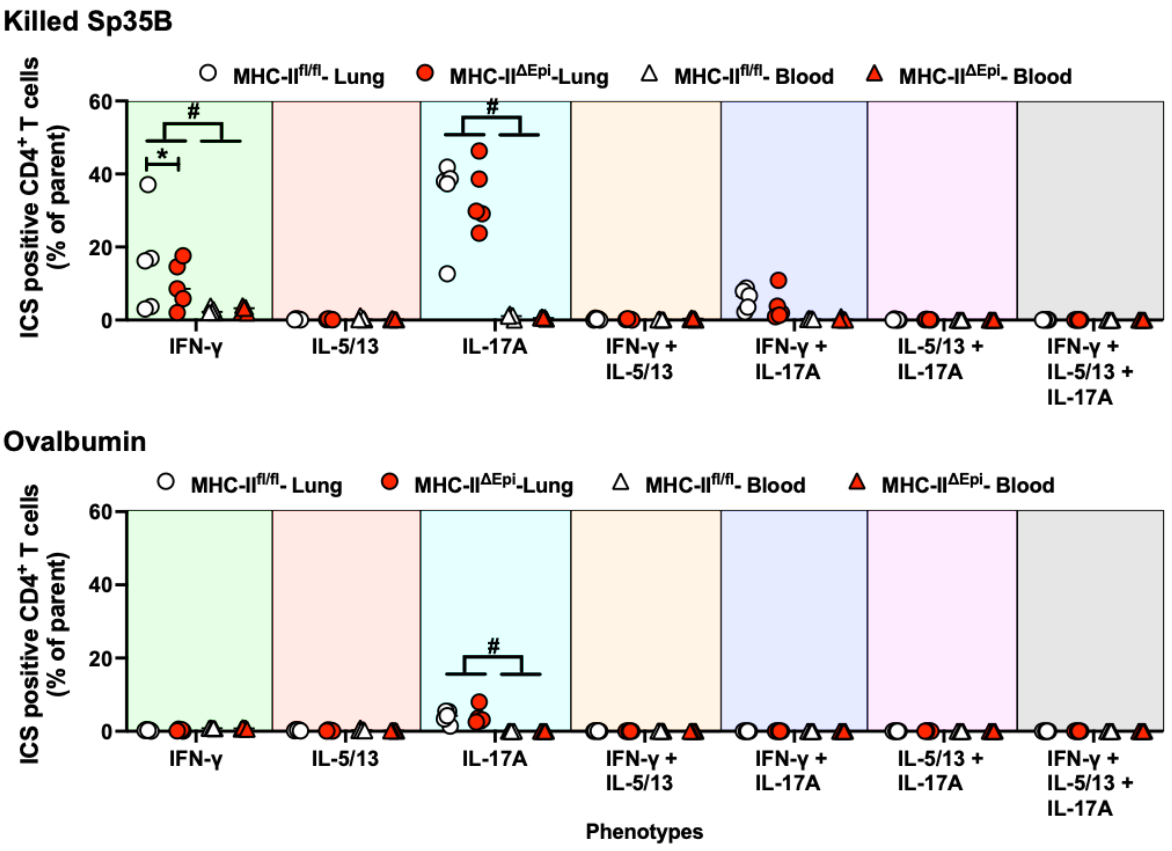

Supplementary Figure. 12: $CD4^+$ T_{RM} cells in MHC-$II^{\Delta Epi}$ mice are *Spn*-specific and show muted T_H1 responses. Intracellular cytokine staining (ICS) profile of lung (i.v.$CD45.2^-$) and blood (i.v.$CD45.2^+$) $CD4^+$ T cells isolated from *Spn*- experienced MHC-$II^{fl/fl}$ and MHC-$II^{\Delta Epi}$ lungs on day 105 and stimulated with beta-propiolactone killed Sp35B or ovalbumin as irrelevant antigen *ex vivo*, two-way ANOVA with two-stage step-up method of Benjamini, Krieger and Yekutieli to correct for multiple comparisons. *p*-value: $^{\#}\leq 0.05$ comparison between lung and blood; $^{*}\leq 0.05$ genotype-dependent comparison within lungs; $^{\Phi}\leq 0.05$ genotype-dependent comparison within blood. All data have $n\geq5$ mice, 2 independent experiments. All data are presented as mean ± SEM.

Supplementary Fig. 13

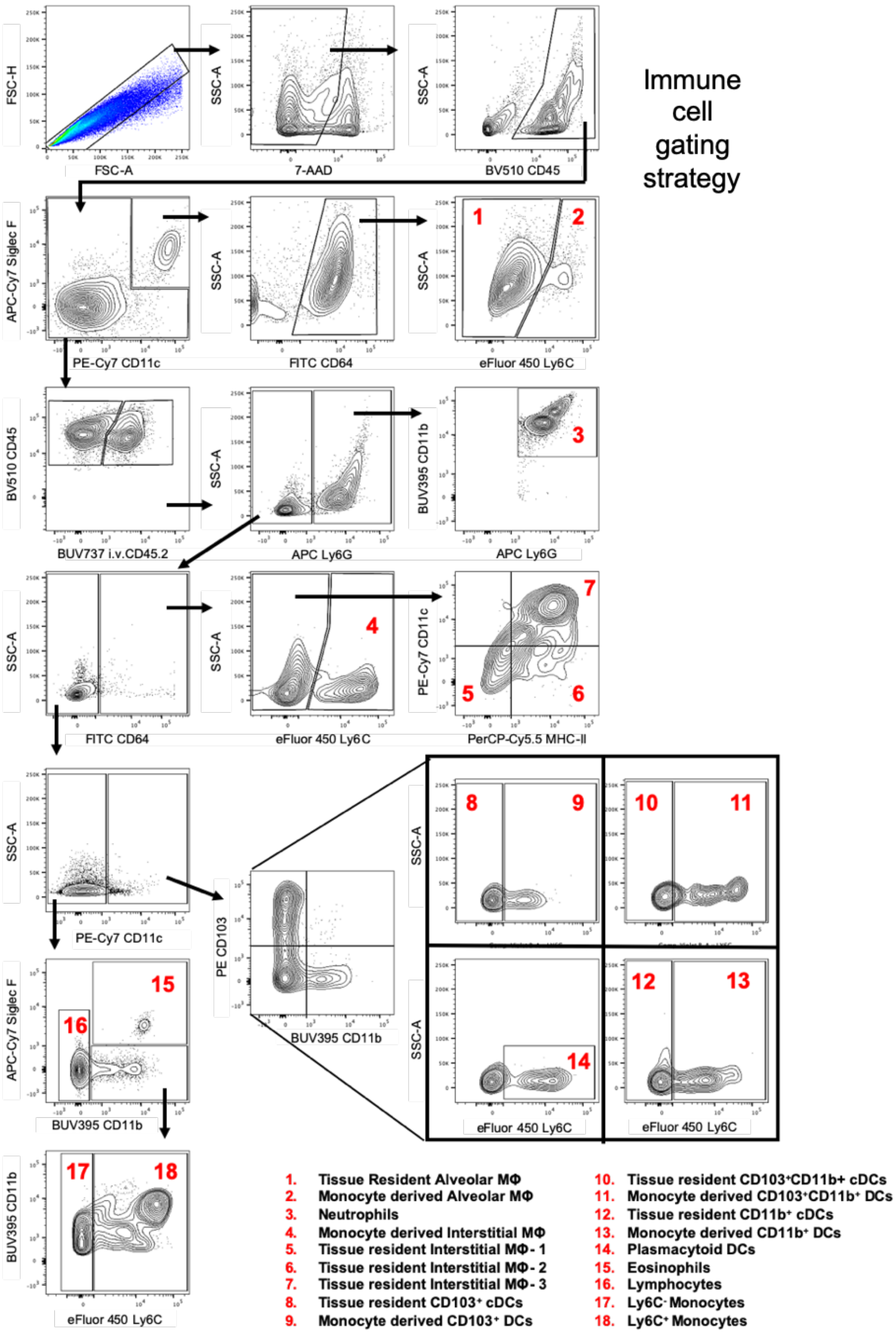

Supplementary Figure. 13: Gating strategy for identification of distinct myeloid cell types by flowcytometry.

Supplementary Fig. 14

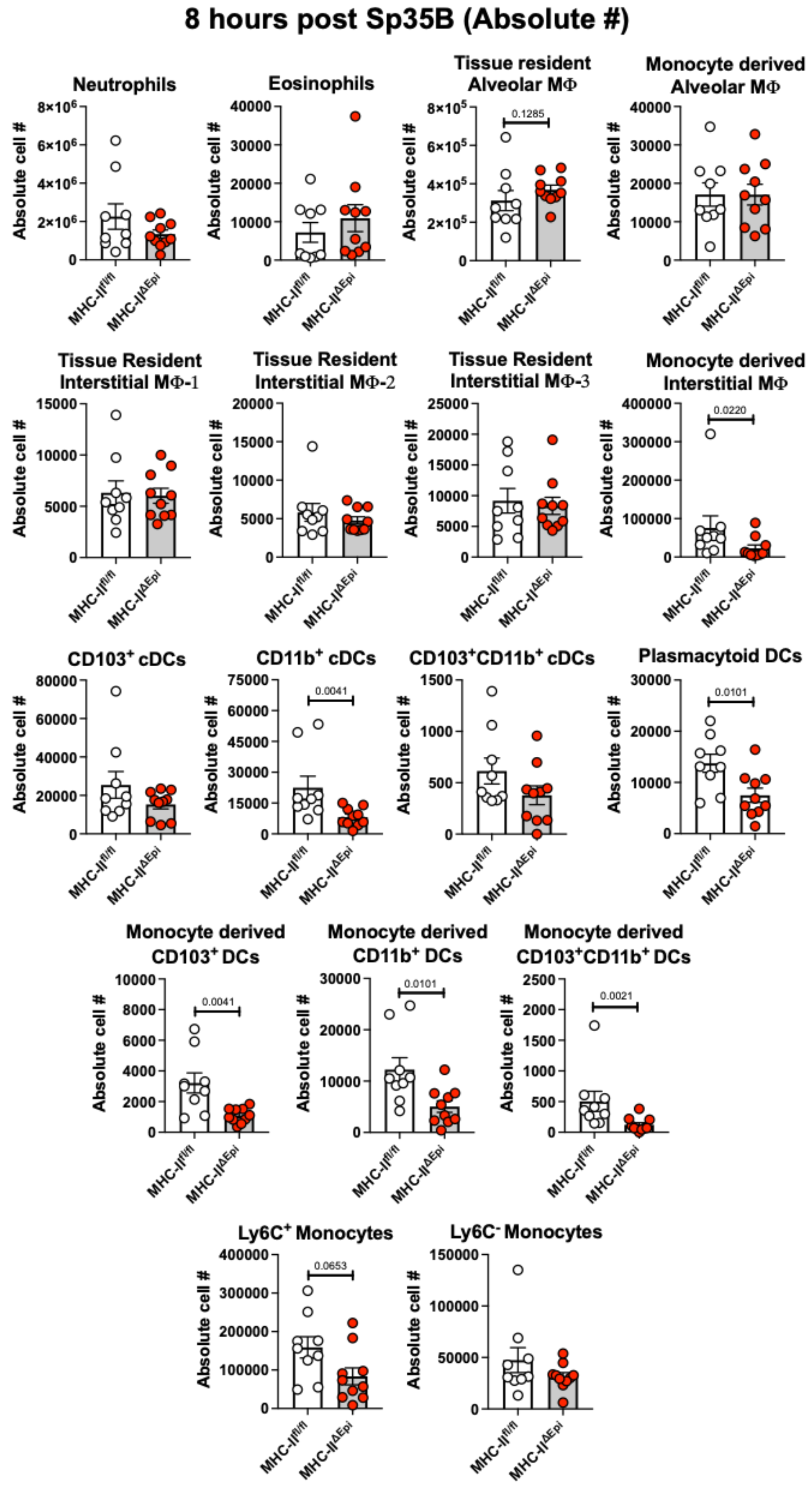

Supplementary Figure. 14: LEC MHC-II regulates barrier immunity. Absolute numbers of major lung (i.v. $CD45.2^{-}$) myeloid cell populations identified in MHC-$II^{fl/fl}$ and MHC-$II^{\Delta Epi}$ lungs 8hpi with Sp35B, two-tailed Mann-Whitney test. All experiments have n≥8 mice, 2 independent experiments. All data are presented as mean ± SEM. Note, the mice used in these experiments belonged to both sexes and were of various sizes at the experiment end-point (~6-8 months old at euthanasia). This was reflected in differences in lung sizes and hence, the frequency data in **Fig. 5c** and absolute numbers herein are both used to draw conclusions.

Supplementary Fig. 15

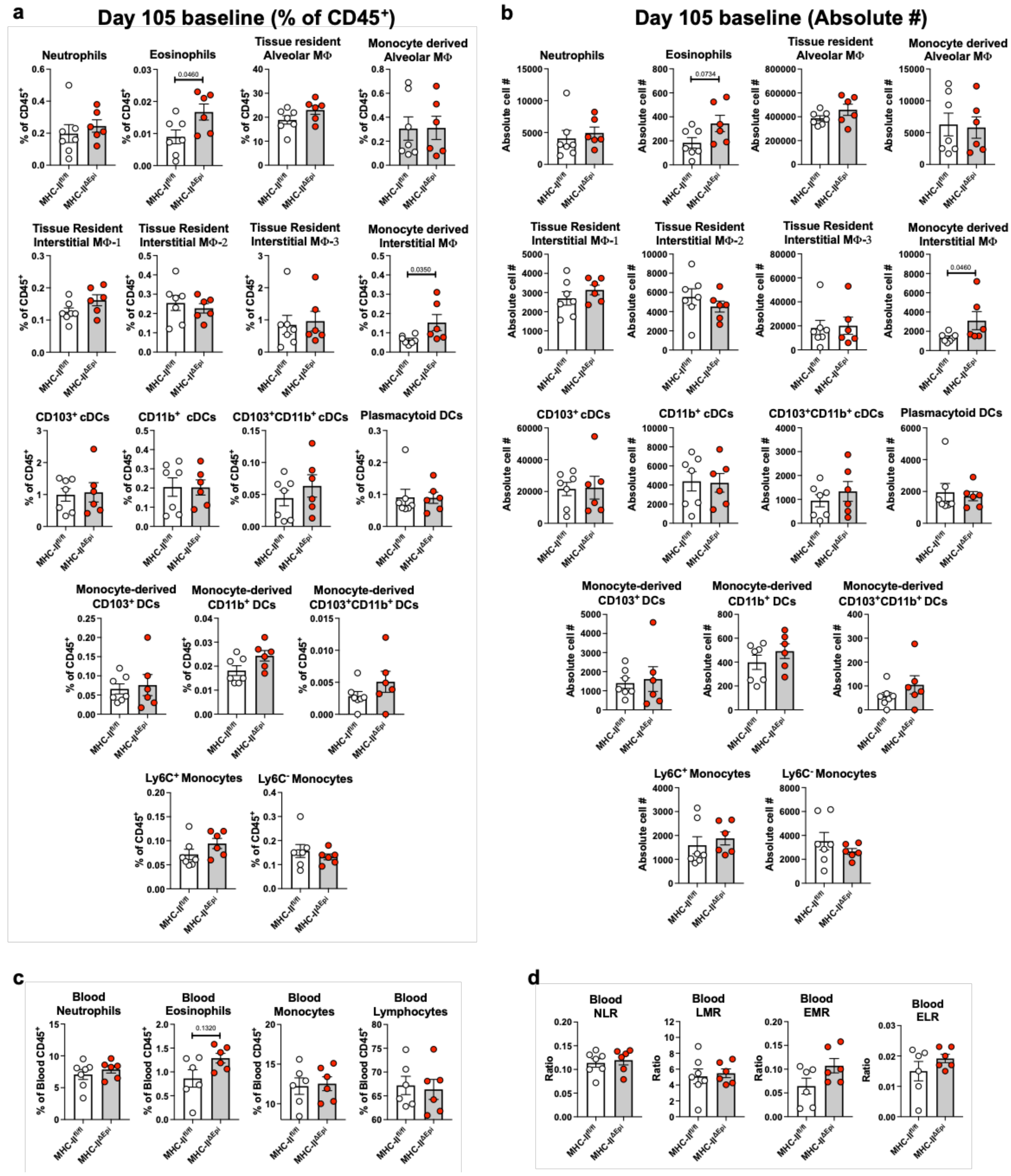

Supplementary Figure. 15: Myeloid innate immune landscape of MHC-II$^{fl/fl}$ and MHC-II$^{\Delta Epi}$ lungs on day 105. a. Frequencies and **b.** Absolute numbers of major lung (i.v. CD45.2^{-}) myeloid cell populations identified in MHC-II$^{fl/fl}$ and MHC-II$^{\Delta Epi}$ lungs on day 105, two-tailed Mann-Whitney test. Note, the mice used in these experiments belonged to both sexes and were of various sizes at the experiment end-point (~6-8 months old at

euthanasia). This was reflected in differences in lung sizes and hence, the frequency and absolute numbers herein are both used to draw conclusions. **c.** Frequencies of intravascular (i.v.CD45.2$^+$) immune cells depicted as fraction of i.v.CD45.2$^+$ cells on day 105, two-tailed Mann-Whitney test. **d.** Blood neutrophil-to-lymphocyte ratio (NLR), lymphocyte-to-monocyte ratio (LMR), eosinophil-to-monocyte ratio (EMR) and eosinophil-to-lymphocyte ratio (ELR) in i.v.CD45.2$^+$ fraction of lungs on day 105, two-tailed Mann-Whitney test. All experiments have n≥6 mice, 2 independent experiments. All data are presented as mean ± SEM.

Supplementary Fig. 16

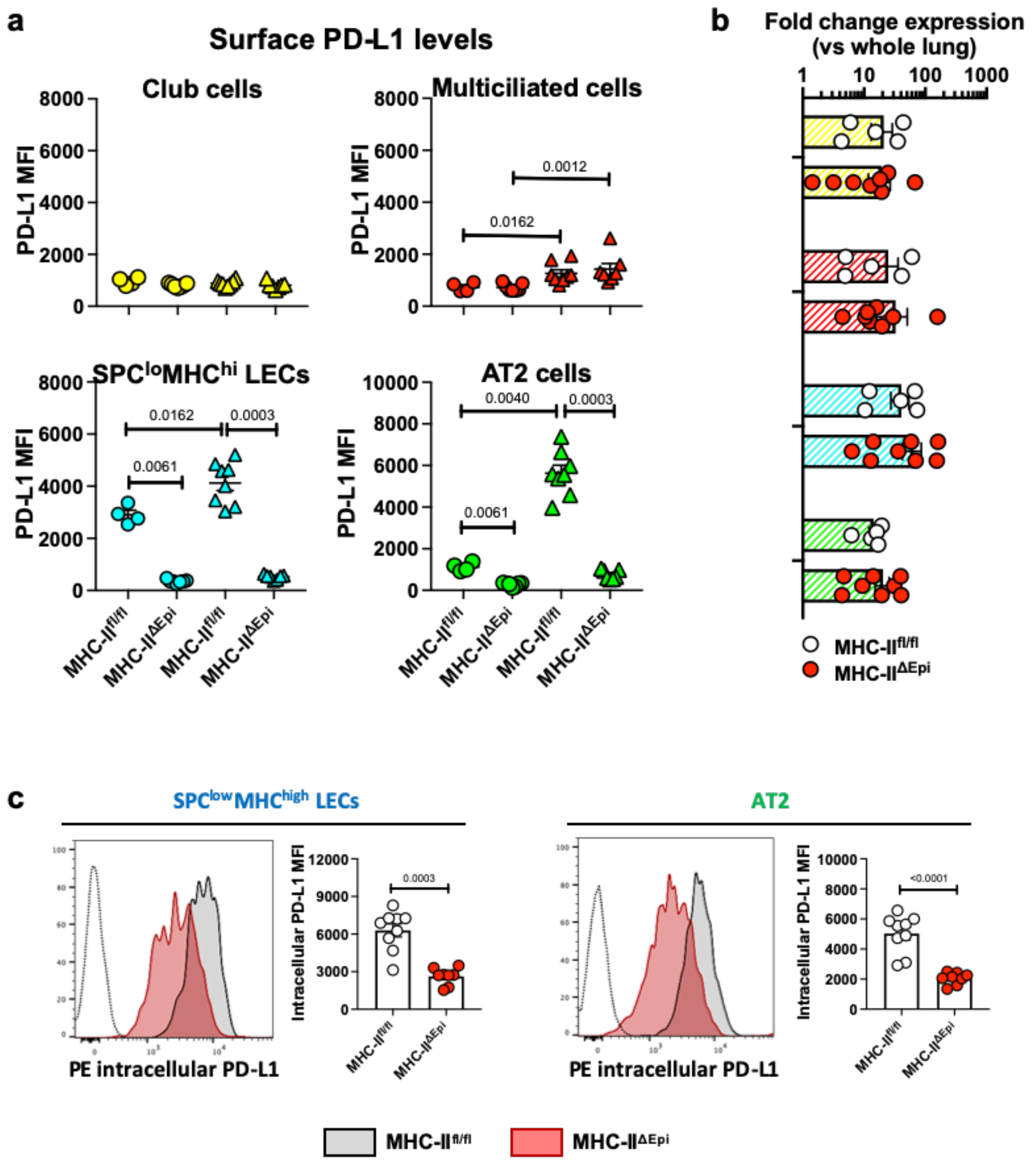

Supplementary Figure. 16: LEC PD-L1 requires MHC-II expression for stability and cell surface display. a. Quantification for surface PD-L1 on LECs from naïve (circles) and Sp19F infected (48hpi, triangles) MHC-II$^{fl/fl}$ and MHC-II$^{\Delta Epi}$ mice, two-tailed Mann-Whitney test. **b.** mRNA levels of PD-L1 transcripts in sorted LECs from Sp19F-infected MHC-II$^{fl/fl}$ and MHC-II$^{\Delta Epi}$ mice 48hpi. Sorting strategy in Supplementary **Figure.2c.c.** Histogram and quantification for intracellular native PD-L1 in alveolar LECs isolated from Sp19F-infected MHC-II$^{fl/fl}$ and MHC-II$^{\Delta Epi}$ mice 48hpi, two-tailed Mann-Whitney test. All experiments have n≥4 mice, 2 independent experiments. All data are presented as mean ± SEM.

Supplementary Fig. 17

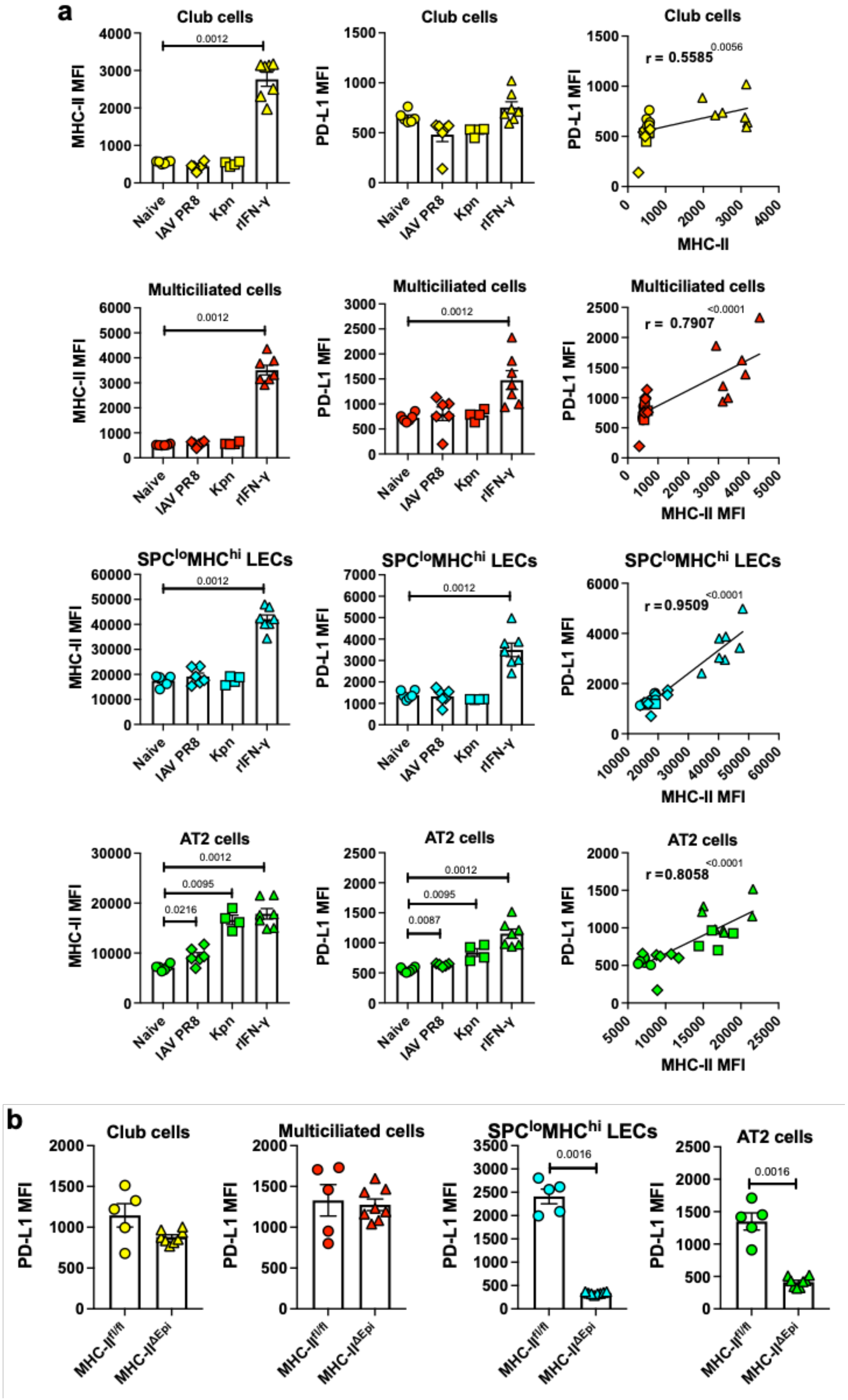

Supplementary Figure. 17: Stable coupling of MHC-II and PD-L1 in LECs is a broadly applicable phenomenon. a. Quantification of surface MHC-II, PD-L1 and scatterplot correlating levels of these molecules on LECs from naïve (circles), Influenza A virus PR8 infected (72hpi, diamonds), *Klebsiella pneumoniae* infected (48hpi, squares) and recombinant IFN-γ treated (24hpi, triangles) C57BL/6J WT mice. Pairwise comparisons were performed by two-tailed Mann-Whitney test. Two-tailed Pearson's correlation coefficient (r) and statistical significance denoted. **b.** Quantification for surface PD-L1 on LECs from naïve (circles) and recombinant IFN-γ treated (24hpi, triangles) MHC-II$^{fl/fl}$ and MHC-II$^{\Delta Epi}$ mice, two-tailed Mann-Whitney test. All experiments have n≥4 mice, 2 independent experiments. All data are presented as mean ± SEM.

Supplementary Fig.18

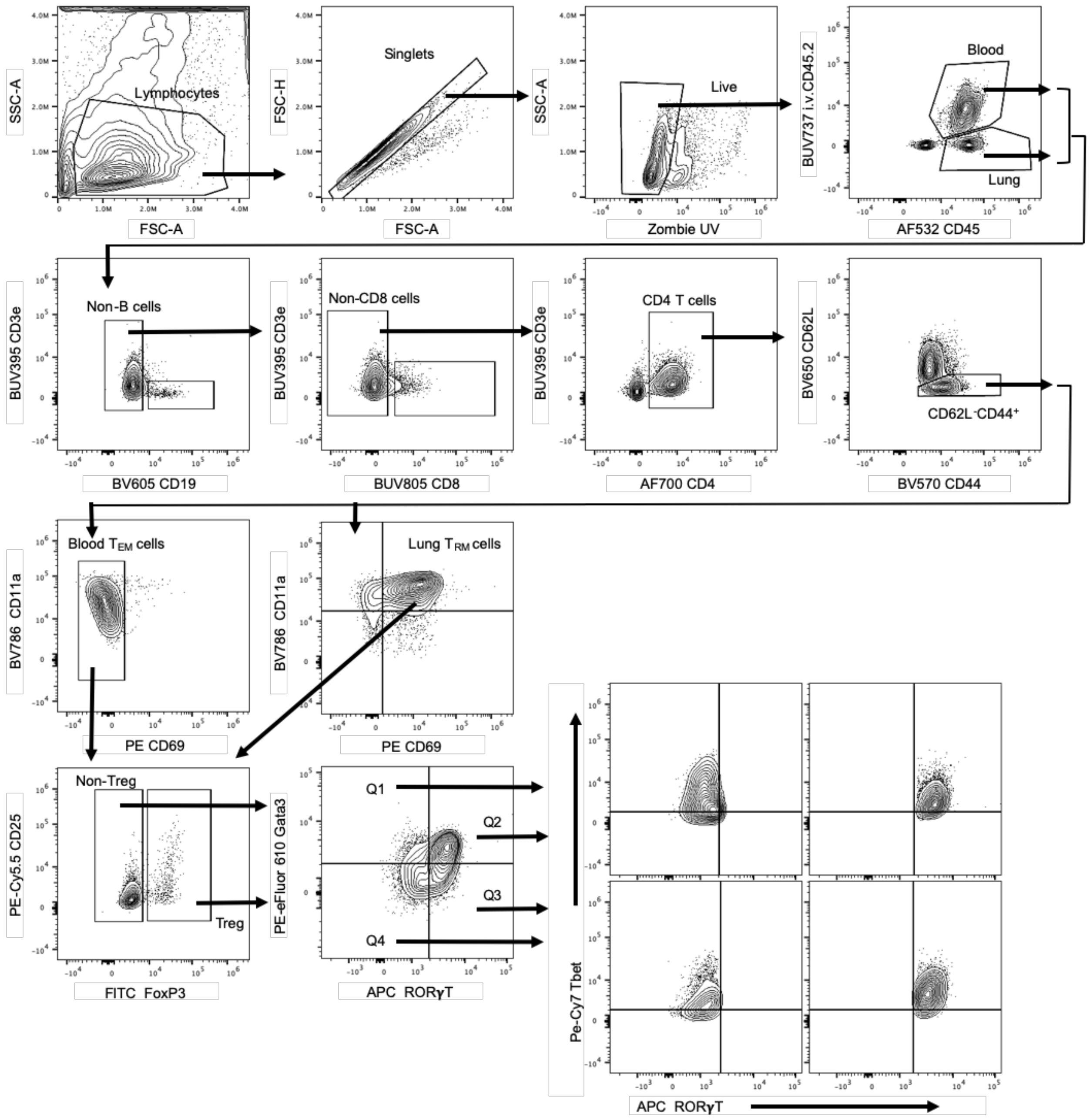

Supplementary Figure. 18: Manual gating strategy for identification of distinct lung CD4⁺ T_{RM} cell and blood CD4⁺ T_{EM} cell lineages in PD-1 knockout mice.

Supplementary Fig.19

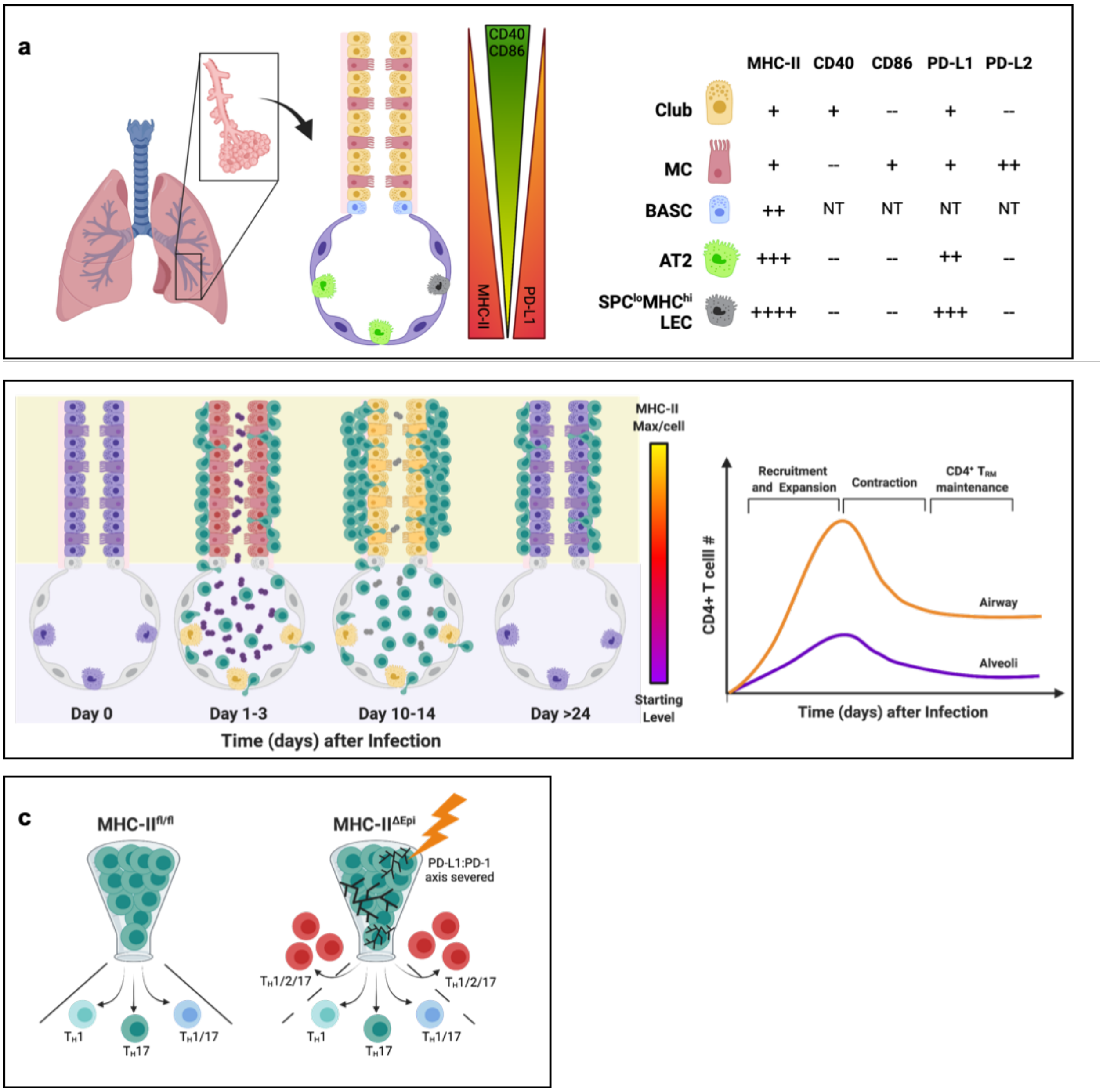

Supplementary Figure. 19: Lung epithelial cell (LEC) antigen presentation governs CD4^{+} T$_{RM}$ cell biology. a. The lung contains repiratory units in which conducting airways end in alveoli. Predominant LECs in conducting airways are club cells (*yellow*) and multiciliated cells (MC, *red*). Bronchialveolar stem cells (BASC, *blue*) are found at the junction between airways and the alveoli, which contain alveolar epithelial type 1 cells (AT1, *purple*), alveolar epithelial type 2 cells (AT2, *green*), and a previously unappreciated alveolar SPC^{low}MHC^{high} cell (*gray*). The three triangles depict trends for MHC-II and related molecules exhibiting a graded expression across these compartments. Relative surface expression for each indicated signal (compared to other LECs in the figure) is represented by increasing numbers of plus signs. NT, not tested. **b.** Lung epithelial MHC-

II expression is temporally dynamic after infection, in association with $CD4^+$ T cell accumulation that persists after resolution as T_{RM} cells around airways. Heat map depicts coloration for LEC MHC-II levels, normalized to baseline for each cell. MHC-II dynamics were not measured for AT1 and BASC. The co-stimulatory and co-inhibitory molecules also show dynamic expression, detailed in the study but not depicted in the summary figure. **c.** LEC MHC-II instructs lung $CD4^+$ T_{RM} cells by restraining their phenotypes and later responses to stimulation, via LEC PD-L1 signaling to PD-1 on T cells. The loss of LEC MHC-II removes the PD-L1 signal and results in aberrant multipotent T_{RM} cells in the lung. Figures created with BioRender.com.

SUPPLEMENTARY TABLES

Supplementary Table 1: Statistics for MHC-II levels on distinct LECs (Related to Fig. 2g)

Two-Way ANOVA	Summary	*p* value	Two-Way ANOVA	Summary	*p* value
Day 0			Day 10		
AT2 vs. Multiciliated	****	<0.0001	AT2 vs. Multiciliated	****	<0.0001
AT2 vs. Club	****	<0.0001	AT2 vs. Club	****	<0.0001
AT2 vs. SPClowMHChigh	****	<0.0001	AT2 vs. SPClowMHChigh	****	<0.0001
AT2 vs. Transitional	****	<0.0001	AT2 vs. Transitional	****	<0.0001
Multiciliated vs. Club	ns	0.9943	Multiciliated vs. Club	ns	0.9566
Multiciliated vs. SPClowMHChigh	****	<0.0001	Multiciliated vs. SPClowMHChigh	****	<0.0001
Multiciliated vs. Transitional	ns	0.9457	Multiciliated vs. Transitional	ns	0.9507
Club vs. SPClowMHChigh	****	<0.0001	Club vs. SPClowMHChigh	****	<0.0001
Club vs. Transitional	ns	0.9401	Club vs. Transitional	ns	0.9075
SPClowMHChigh vs. Transitional	****	<0.0001	SPClowMHChigh vs. Transitional	****	<0.0001
Day 1			Day 14		
AT2 vs. Multiciliated	****	<0.0001	AT2 vs. Multiciliated	****	<0.0001
AT2 vs. Club	****	<0.0001	AT2 vs. Club	****	<0.0001
AT2 vs. SPClowMHChigh	****	<0.0001	AT2 vs. SPClowMHChigh	****	<0.0001
AT2 vs. Transitional	****	<0.0001	AT2 vs. Transitional	****	<0.0001
Multiciliated vs. Club	ns	0.9178	Multiciliated vs. Club	ns	0.9623
Multiciliated vs. SPClowMHChigh	****	<0.0001	Multiciliated vs. SPClowMHChigh	****	<0.0001
Multiciliated vs. Transitional	ns	0.8905	Multiciliated vs. Transitional	ns	0.9247
Club vs. SPClowMHChigh	****	<0.0001	Club vs. SPClowMHChigh	****	<0.0001
Club vs. Transitional	ns	0.9725	Club vs. Transitional	ns	0.9623
SPClowMHChigh vs. Transitional	****	<0.0001	SPClowMHChigh vs. Transitional	****	<0.0001
Day 3			Day 24		
AT2 vs. Multiciliated	****	<0.0001	AT2 vs. Multiciliated	****	<0.0001
AT2 vs. Club	****	<0.0001	AT2 vs. Club	****	<0.0001
AT2 vs. SPClowMHChigh	ns	0.1524	AT2 vs. SPClowMHChigh	****	<0.0001
AT2 vs. Transitional	****	<0.0001	AT2 vs. Transitional	****	<0.0001
Multiciliated vs. Club	ns	0.8799	Multiciliated vs. Club	ns	0.9498
Multiciliated vs. SPClowMHChigh	****	<0.0001	Multiciliated vs. SPClowMHChigh	****	<0.0001
Multiciliated vs. Transitional	ns	0.8708	Multiciliated vs. Transitional	ns	0.9261
Club vs. SPClowMHChigh	****	<0.0001	Club vs. SPClowMHChigh	****	<0.0001
Club vs. Transitional	ns	0.9907	Club vs. Transitional	ns	0.9762
SPClowMHChigh vs. Transitional	****	<0.0001	SPClowMHChigh vs. Transitional	****	<0.0001
Day 7			Day 35		
AT2 vs. Multiciliated	****	<0.0001	AT2 vs. Multiciliated	****	<0.0001
AT2 vs. Club	****	<0.0001	AT2 vs. Club	****	<0.0001
AT2 vs. SPClowMHChigh	****	<0.0001	AT2 vs. SPClowMHChigh	****	<0.0001
AT2 vs. Transitional	****	<0.0001	AT2 vs. Transitional	****	<0.0001
Multiciliated vs. Club	ns	0.9157	Multiciliated vs. Club	ns	0.975
Multiciliated vs. SPClowMHChigh	****	<0.0001	Multiciliated vs. SPClowMHChigh	****	<0.0001
Multiciliated vs. Transitional	ns	0.8991	Multiciliated vs. Transitional	ns	0.936
Club vs. SPClowMHChigh	****	<0.0001	Club vs. SPClowMHChigh	****	<0.0001
Club vs. Transitional	ns	0.9833	Club vs. Transitional	ns	0.9609
SPClowMHChigh vs. Transitional	****	<0.0001	SPClowMHChigh vs. Transitional	****	<0.0001
Day 8					
AT2 vs. Multiciliated	****	<0.0001			
AT2 vs. Club	****	<0.0001			
AT2 vs. SPClowMHChigh	****	<0.0001			
AT2 vs. Transitional	****	<0.0001			
Multiciliated vs. Club	ns	0.7806			
Multiciliated vs. SPClowMHChigh	****	<0.0001			
Multiciliated vs. Transitional	ns	0.6541			
Club vs. SPClowMHChigh	****	<0.0001			
Club vs. Transitional	ns	0.8653			
SPClowMHChigh vs. Transitional	****	<0.0001			

Supplementary Table 2: Statistics for CD40 levels on distinct LECs (Related to Fig. 2h)

Two-Way ANOVA	Summary	*p* value		Two-Way ANOVA	Summary	*p* value
Day 0				Day 10		
AT2 vs. Multiciliated	ns	0.6087		AT2 vs. Multiciliated	ns	0.8521
AT2 vs. Club	****	<0.0001		AT2 vs. Club	****	<0.0001
AT2 vs. SPClowMHChigh	*	0.0143		AT2 vs. SPClowMHChigh	*	0.0334
AT2 vs. Transitional	**	0.0036		AT2 vs. Transitional	*	0.0334
Multiciliated vs. Club	****	<0.0001		Multiciliated vs. Club	****	<0.0001
Multiciliated vs. SPClowMHChigh	ns	0.0516		Multiciliated vs. SPClowMHChigh	*	0.0209
Multiciliated vs. Transitional	*	0.0158		Multiciliated vs. Transitional	*	0.0208
Club vs. SPClowMHChigh	****	<0.0001		Club vs. SPClowMHChigh	**	0.0043
Club vs. Transitional	****	<0.0001		Club vs. Transitional	**	0.0043
SPClowMHChigh vs. Transitional	ns	0.6345		SPClowMHChigh vs. Transitional	ns	0.9994
Day 1				Day 14		
AT2 vs. Multiciliated	ns	0.837		AT2 vs. Multiciliated	ns	0.9421
AT2 vs. Club	****	<0.0001		AT2 vs. Club	****	<0.0001
AT2 vs. SPClowMHChigh	ns	0.0729		AT2 vs. SPClowMHChigh	*	0.0394
AT2 vs. Transitional	**	0.0015		AT2 vs. Transitional	*	0.0371
Multiciliated vs. Club	****	<0.0001		Multiciliated vs. Club	****	<0.0001
Multiciliated vs. SPClowMHChigh	*	0.0459		Multiciliated vs. SPClowMHChigh	*	0.033
Multiciliated vs. Transitional	***	0.0008		Multiciliated vs. Transitional	*	0.0311
Club vs. SPClowMHChigh	****	<0.0001		Club vs. SPClowMHChigh	*	0.0293
Club vs. Transitional	****	<0.0001		Club vs. Transitional	*	0.0312
SPClowMHChigh vs. Transitional	ns	0.1613		SPClowMHChigh vs. Transitional	ns	0.9802
Day 3				Day 24		
AT2 vs. Multiciliated	ns	0.7836		AT2 vs. Multiciliated	ns	0.2666
AT2 vs. Club	****	<0.0001		AT2 vs. Club	****	<0.0001
AT2 vs. SPClowMHChigh	*	0.0487		AT2 vs. SPClowMHChigh	*	0.0198
AT2 vs. Transitional	***	0.0007		AT2 vs. Transitional	*	0.0231
Multiciliated vs. Club	****	<0.0001		Multiciliated vs. Club	****	<0.0001
Multiciliated vs. SPClowMHChigh	*	0.025		Multiciliated vs. SPClowMHChigh	ns	0.2188
Multiciliated vs. Transitional	***	0.0003		Multiciliated vs. Transitional	ns	0.242
Club vs. SPClowMHChigh	****	<0.0001		Club vs. SPClowMHChigh	****	<0.0001
Club vs. Transitional	****	<0.0001		Club vs. Transitional	****	<0.0001
SPClowMHChigh vs. Transitional	ns	0.1481		SPClowMHChigh vs. Transitional	ns	0.9521
Day 7				Day 35		
AT2 vs. Multiciliated	ns	0.822		AT2 vs. Multiciliated	ns	0.5769
AT2 vs. Club	****	<0.0001		AT2 vs. Club	****	<0.0001
AT2 vs. SPClowMHChigh	*	0.0152		AT2 vs. SPClowMHChigh	*	0.0232
AT2 vs. Transitional	ns	0.0984		AT2 vs. Transitional	*	0.0235
Multiciliated vs. Club	***	0.0002		Multiciliated vs. Club	****	<0.0001
Multiciliated vs. SPClowMHChigh	*	0.0273		Multiciliated vs. SPClowMHChigh	ns	0.0857
Multiciliated vs. Transitional	ns	0.1529		Multiciliated vs. Transitional	ns	0.0864
Club vs. SPClowMHChigh	ns	0.1296		Club vs. SPClowMHChigh	****	<0.0001
Club vs. Transitional	*	0.0219		Club vs. Transitional	****	<0.0001
SPClowMHChigh vs. Transitional	ns	0.432		SPClowMHChigh vs. Transitional	ns	0.9971
Day 8						
AT2 vs. Multiciliated	ns	0.5647				
AT2 vs. Club	***	0.0002				
AT2 vs. SPClowMHChigh	*	0.0257				
AT2 vs. Transitional	ns	0.1327				
Multiciliated vs. Club	****	<0.0001				
Multiciliated vs. SPClowMHChigh	**	0.0052				
Multiciliated vs. Transitional	*	0.0381				
Club vs. SPClowMHChigh	ns	0.1105				
Club vs. Transitional	*	0.0202				
SPClowMHChigh vs. Transitional	ns	0.4621				

Supplementary Table 3: Statistics for CD86 levels on distinct LECs (Related to Fig. 2i)

Two-Way ANOVA	Summary	*p* value		Two-Way ANOVA	Summary	*p* value
Day 0				Day 10		
AT2 vs. Multiciliated	****	<0.0001		AT2 vs. Multiciliated	****	<0.0001
AT2 vs. Club	*	0.0186		AT2 vs. Club	ns	0.0864
AT2 vs. SPClowMHChigh	ns	0.2466		AT2 vs. SPClowMHChigh	ns	0.1964
AT2 vs. Transitional	****	<0.0001		AT2 vs. Transitional	***	0.0009
Multiciliated vs. Club	****	<0.0001		Multiciliated vs. Club	****	<0.0001
Multiciliated vs. SPClowMHChigh	****	<0.0001		Multiciliated vs. SPClowMHChigh	****	<0.0001
Multiciliated vs. Transitional	***	0.0001		Multiciliated vs. Transitional	**	0.0021
Club vs. SPClowMHChigh	ns	0.2283		Club vs. SPClowMHChigh	ns	0.67
Club vs. Transitional	*	0.0121		Club vs. Transitional	ns	0.099
SPClowMHChigh vs. Transitional	***	0.0002		SPClowMHChigh vs. Transitional	*	0.0384
Day 1				Day 14		
AT2 vs. Multiciliated	****	<0.0001		AT2 vs. Multiciliated	****	<0.0001
AT2 vs. Club	ns	0.4043		AT2 vs. Club	ns	0.1124
AT2 vs. SPClowMHChigh	ns	0.8893		AT2 vs. SPClowMHChigh	ns	0.2874
AT2 vs. Transitional	****	<0.0001		AT2 vs. Transitional	***	0.0004
Multiciliated vs. Club	****	<0.0001		Multiciliated vs. Club	****	<0.0001
Multiciliated vs. SPClowMHChigh	****	<0.0001		Multiciliated vs. SPClowMHChigh	****	<0.0001
Multiciliated vs. Transitional	****	<0.0001		Multiciliated vs. Transitional	****	<0.0001
Club vs. SPClowMHChigh	ns	0.3306		Club vs. SPClowMHChigh	ns	0.5984
Club vs. Transitional	**	0.001		Club vs. Transitional	*	0.0438
SPClowMHChigh vs. Transitional	****	<0.0001		SPClowMHChigh vs. Transitional	*	0.0113
Day 3				Day 24		
AT2 vs. Multiciliated	****	<0.0001		AT2 vs. Multiciliated	****	<0.0001
AT2 vs. Club	ns	0.1353		AT2 vs. Club	*	0.0396
AT2 vs. SPClowMHChigh	ns	0.4907		AT2 vs. SPClowMHChigh	ns	0.1901
AT2 vs. Transitional	***	0.0004		AT2 vs. Transitional	****	<0.0001
Multiciliated vs. Club	****	<0.0001		Multiciliated vs. Club	****	<0.0001
Multiciliated vs. SPClowMHChigh	****	<0.0001		Multiciliated vs. SPClowMHChigh	****	<0.0001
Multiciliated vs. Transitional	****	<0.0001		Multiciliated vs. Transitional	****	<0.0001
Club vs. SPClowMHChigh	ns	0.4195		Club vs. SPClowMHChigh	ns	0.4505
Club vs. Transitional	*	0.0364		Club vs. Transitional	*	0.0149
SPClowMHChigh vs. Transitional	**	0.0039		SPClowMHChigh vs. Transitional	**	0.0015
Day 7				Day 35		
AT2 vs. Multiciliated	****	<0.0001		AT2 vs. Multiciliated	****	<0.0001
AT2 vs. Club	ns	0.0675		AT2 vs. Club	**	0.0093
AT2 vs. SPClowMHChigh	ns	0.7489		AT2 vs. SPClowMHChigh	ns	0.1463
AT2 vs. Transitional	****	<0.0001		AT2 vs. Transitional	****	<0.0001
Multiciliated vs. Club	****	<0.0001		Multiciliated vs. Club	****	<0.0001
Multiciliated vs. SPClowMHChigh	****	<0.0001		Multiciliated vs. SPClowMHChigh	****	<0.0001
Multiciliated vs. Transitional	****	<0.0001		Multiciliated vs. Transitional	****	<0.0001
Club vs. SPClowMHChigh	*	0.032		Club vs. SPClowMHChigh	ns	0.2443
Club vs. Transitional	**	0.009		Club vs. Transitional	**	0.0024
SPClowMHChigh vs. Transitional	****	<0.0001		SPClowMHChigh vs. Transitional	****	<0.0001
Day 8						
AT2 vs. Multiciliated	****	<0.0001				
AT2 vs. Club	ns	0.1299				
AT2 vs. SPClowMHChigh	**	0.0039				
AT2 vs. Transitional	ns	0.2814				
Multiciliated vs. Club	****	<0.0001				
Multiciliated vs. SPClowMHChigh	****	<0.0001				
Multiciliated vs. Transitional	****	<0.0001				
Club vs. SPClowMHChigh	ns	0.1644				
Club vs. Transitional	**	0.0099				
SPClowMHChigh vs. Transitional	****	<0.0001				

Supplementary Table 4: Statistics for ICAM1 levels on distinct LECs (Related to Fig. 2j)

Two-Way ANOVA	Summary	*p* value	Two-Way ANOVA	Summary	*p* value
Day 0			Day 10		
AT2 vs. Multiciliated	ns	0.1652	AT2 vs. Multiciliated	**	0.0057
AT2 vs. Club	*	0.041	AT2 vs. Club	****	<0.0001
AT2 vs. SPClowMHChigh	ns	0.9359	AT2 vs. SPClowMHChigh	**	0.0029
AT2 vs. Transitional	*	0.0337	AT2 vs. Transitional	****	<0.0001
Multiciliated vs. Club	ns	0.5083	Multiciliated vs. Club	*	0.0142
Multiciliated vs. SPClowMHChigh	ns	0.1909	Multiciliated vs. SPClowMHChigh	ns	0.8272
Multiciliated vs. Transitional	ns	0.4577	Multiciliated vs. Transitional	*	0.0376
Club vs. SPClowMHChigh	*	0.0495	Club vs. SPClowMHChigh	*	0.0253
Club vs. Transitional	ns	0.9353	Club vs. Transitional	ns	0.7057
SPClowMHChigh vs. Transitional	*	0.041	SPClowMHChigh vs. Transitional	ns	0.0623
Day 1			Day 14		
AT2 vs. Multiciliated	****	<0.0001	AT2 vs. Multiciliated	ns	0.0752
AT2 vs. Club	****	<0.0001	AT2 vs. Club	*	0.0136
AT2 vs. SPClowMHChigh	****	<0.0001	AT2 vs. SPClowMHChigh	ns	0.3322
AT2 vs. Transitional	****	<0.0001	AT2 vs. Transitional	**	0.0085
Multiciliated vs. Club	****	<0.0001	Multiciliated vs. Club	ns	0.4844
Multiciliated vs. SPClowMHChigh	**	0.0042	Multiciliated vs. SPClowMHChigh	ns	0.4155
Multiciliated vs. Transitional	****	<0.0001	Multiciliated vs. Transitional	ns	0.3871
Club vs. SPClowMHChigh	****	<0.0001	Club vs. SPClowMHChigh	ns	0.1309
Club vs. Transitional	ns	0.8997	Club vs. Transitional	ns	0.8682
SPClowMHChigh vs. Transitional	****	<0.0001	SPClowMHChigh vs. Transitional	ns	0.0939
Day 3			Day 24		
AT2 vs. Multiciliated	****	<0.0001	AT2 vs. Multiciliated	ns	0.1224
AT2 vs. Club	****	<0.0001	AT2 vs. Club	*	0.024
AT2 vs. SPClowMHChigh	****	<0.0001	AT2 vs. SPClowMHChigh	ns	0.5345
AT2 vs. Transitional	****	<0.0001	AT2 vs. Transitional	*	0.0262
Multiciliated vs. Club	****	<0.0001	Multiciliated vs. Club	ns	0.4708
Multiciliated vs. SPClowMHChigh	ns	0.6272	Multiciliated vs. SPClowMHChigh	ns	0.3542
Multiciliated vs. Transitional	***	0.0007	Multiciliated vs. Transitional	ns	0.4924
Club vs. SPClowMHChigh	***	0.0005	Club vs. SPClowMHChigh	ns	0.1002
Club vs. Transitional	ns	0.5775	Club vs. Transitional	ns	0.9724
SPClowMHChigh vs. Transitional	**	0.0033	SPClowMHChigh vs. Transitional	ns	0.1075
Day 7			Day 35		
AT2 vs. Multiciliated	*	0.0438	AT2 vs. Multiciliated	ns	0.072
AT2 vs. Club	*	0.018	AT2 vs. Club	*	0.0169
AT2 vs. SPClowMHChigh	ns	0.5085	AT2 vs. SPClowMHChigh	ns	0.7329
AT2 vs. Transitional	*	0.0111	AT2 vs. Transitional	*	0.0216
Multiciliated vs. Club	ns	0.7231	Multiciliated vs. Club	ns	0.5491
Multiciliated vs. SPClowMHChigh	ns	0.1734	Multiciliated vs. SPClowMHChigh	ns	0.144
Multiciliated vs. Transitional	ns	0.594	Multiciliated vs. Transitional	ns	0.6137
Club vs. SPClowMHChigh	ns	0.0867	Club vs. SPClowMHChigh	*	0.04
Club vs. Transitional	ns	0.8581	Club vs. Transitional	ns	0.9248
SPClowMHChigh vs. Transitional	ns	0.0588	SPClowMHChigh vs. Transitional	*	0.0499
Day 8					
AT2 vs. Multiciliated	****	<0.0001			
AT2 vs. Club	****	<0.0001			
AT2 vs. SPClowMHChigh	****	<0.0001			
AT2 vs. Transitional	****	<0.0001			
Multiciliated vs. Club	****	<0.0001			
Multiciliated vs. SPClowMHChigh	ns	0.9576			
Multiciliated vs. Transitional	****	<0.0001			
Club vs. SPClowMHChigh	****	<0.0001			
Club vs. Transitional	ns	0.3448			
SPClowMHChigh vs. Transitional	****	<0.0001			

Supplementary Table 5: Statistics for PD-L1 levels on distinct LECs (Related to Fig. 2k)

Two-Way ANOVA	Summary	*p* value		Two-Way ANOVA	Summary	*p* value
Day 0				Day 10		
AT2 vs. Multiciliated	ns	0.925		AT2 vs. Multiciliated	****	<0.0001
AT2 vs. Club	ns	0.9283		AT2 vs. Club	****	<0.0001
AT2 vs. SPClowMHChigh	****	<0.0001		AT2 vs. SPClowMHChigh	****	<0.0001
AT2 vs. Transitional	ns	0.8222		AT2 vs. Transitional	****	<0.0001
Multiciliated vs. Club	ns	0.9967		Multiciliated vs. Club	ns	0.0658
Multiciliated vs. SPClowMHChigh	****	<0.0001		Multiciliated vs. SPClowMHChigh	****	<0.0001
Multiciliated vs. Transitional	ns	0.7499		Multiciliated vs. Transitional	ns	0.4216
Club vs. SPClowMHChigh	****	<0.0001		Club vs. SPClowMHChigh	****	<0.0001
Club vs. Transitional	ns	0.7531		Club vs. Transitional	ns	0.2977
SPClowMHChigh vs. Transitional	****	<0.0001		SPClowMHChigh vs. Transitional	****	<0.0001
Day 1				Day 14		
AT2 vs. Multiciliated	*	0.0389		AT2 vs. Multiciliated	****	<0.0001
AT2 vs. Club	***	0.0005		AT2 vs. Club	****	<0.0001
AT2 vs. SPClowMHChigh	****	<0.0001		AT2 vs. SPClowMHChigh	****	<0.0001
AT2 vs. Transitional	***	0.001		AT2 vs. Transitional	****	<0.0001
Multiciliated vs. Club	ns	0.1408		Multiciliated vs. Club	ns	0.5435
Multiciliated vs. SPClowMHChigh	****	<0.0001		Multiciliated vs. SPClowMHChigh	****	<0.0001
Multiciliated vs. Transitional	ns	0.2052		Multiciliated vs. Transitional	ns	0.4976
Club vs. SPClowMHChigh	****	<0.0001		Club vs. SPClowMHChigh	****	<0.0001
Club vs. Transitional	ns	0.8358		Club vs. Transitional	ns	0.9436
SPClowMHChigh vs. Transitional	****	<0.0001		SPClowMHChigh vs. Transitional	****	<0.0001
Day 3				Day 24		
AT2 vs. Multiciliated	****	<0.0001		AT2 vs. Multiciliated	ns	0.6497
AT2 vs. Club	****	<0.0001		AT2 vs. Club	ns	0.2215
AT2 vs. SPClowMHChigh	****	<0.0001		AT2 vs. SPClowMHChigh	****	<0.0001
AT2 vs. Transitional	****	<0.0001		AT2 vs. Transitional	ns	0.165
Multiciliated vs. Club	ns	0.141		Multiciliated vs. Club	ns	0.4415
Multiciliated vs. SPClowMHChigh	****	<0.0001		Multiciliated vs. SPClowMHChigh	****	<0.0001
Multiciliated vs. Transitional	ns	0.2793		Multiciliated vs. Transitional	ns	0.3491
Club vs. SPClowMHChigh	****	<0.0001		Club vs. SPClowMHChigh	****	<0.0001
Club vs. Transitional	ns	0.6949		Club vs. Transitional	ns	0.8674
SPClowMHChigh vs. Transitional	****	<0.0001		SPClowMHChigh vs. Transitional	****	<0.0001
Day 7				Day 35		
AT2 vs. Multiciliated	ns	0.3416		AT2 vs. Multiciliated	ns	0.8647
AT2 vs. Club	*	0.0301		AT2 vs. Club	ns	0.5544
AT2 vs. SPClowMHChigh	****	<0.0001		AT2 vs. SPClowMHChigh	****	<0.0001
AT2 vs. Transitional	*	0.0355		AT2 vs. Transitional	ns	0.4399
Multiciliated vs. Club	ns	0.2199		Multiciliated vs. Club	ns	0.6738
Multiciliated vs. SPClowMHChigh	****	<0.0001		Multiciliated vs. SPClowMHChigh	****	<0.0001
Multiciliated vs. Transitional	ns	0.2462		Multiciliated vs. Transitional	ns	0.547
Club vs. SPClowMHChigh	****	<0.0001		Club vs. SPClowMHChigh	****	<0.0001
Club vs. Transitional	ns	0.9462		Club vs. Transitional	ns	0.8559
SPClowMHChigh vs. Transitional	****	<0.0001		SPClowMHChigh vs. Transitional	****	<0.0001
Day 8						
AT2 vs. Multiciliated	ns	0.6141				
AT2 vs. Club	****	<0.0001				
AT2 vs. SPClowMHChigh	****	<0.0001				
AT2 vs. Transitional	ns	0.1706				
Multiciliated vs. Club	****	<0.0001				
Multiciliated vs. SPClowMHChigh	****	<0.0001				
Multiciliated vs. Transitional	ns	0.3853				
Club vs. SPClowMHChigh	****	<0.0001				
Club vs. Transitional	***	0.0001				
SPClowMHChigh vs. Transitional	****	<0.0001				

Supplementary Table 6: Statistics for PD-L2 levels on distinct LECs (Related to Fig. 2I)

Two-Way ANOVA	Summary	*p* value		Two-Way ANOVA	Summary	*p* value
Day 0				Day 10		
AT2 vs. Multiciliated	****	<0.0001		AT2 vs. Multiciliated	****	<0.0001
AT2 vs. Club	ns	0.0907		AT2 vs. Club	ns	0.0631
AT2 vs. SPClowMHChigh	ns	0.5751		AT2 vs. MHChigh	ns	0.1955
AT2 vs. Transitional	***	0.0003		AT2 vs. Transitional	**	0.0018
Multiciliated vs. Club	**	0.0012		Multiciliated vs. Club	*	0.0124
Multiciliated vs. SPClowMHChigh	****	<0.0001		Multiciliated vs. MHChigh	**	0.0022
Multiciliated vs. Transitional	ns	0.1925		Multiciliated vs. Transitional	ns	0.2177
Club vs. SPClowMHChigh	ns	0.2565		Club vs. MHChigh	ns	0.5694
Club vs. Transitional	*	0.0485		Club vs. Transitional	ns	0.2002
SPClowMHChigh vs. Transitional	**	0.002		MHChigh vs. Transitional	ns	0.065
Day 1				Day 14		
AT2 vs. Multiciliated	**	0.0027		AT2 vs. Multiciliated	****	<0.0001
AT2 vs. Club	****	<0.0001		AT2 vs. Club	*	0.0131
AT2 vs. SPClowMHChigh	****	<0.0001		AT2 vs. SPClowMHChigh	ns	0.2019
AT2 vs. Transitional	****	<0.0001		AT2 vs. Transitional	****	<0.0001
Multiciliated vs. Club	****	<0.0001		Multiciliated vs. Club	****	<0.0001
Multiciliated vs. SPClowMHChigh	****	<0.0001		Multiciliated vs. SPClowMHChigh	****	<0.0001
Multiciliated vs. Transitional	****	<0.0001		Multiciliated vs. Transitional	****	<0.0001
Club vs. SPClowMHChigh	ns	0.2101		Club vs. SPClowMHChigh	ns	0.2235
Club vs. Transitional	**	0.002		Club vs. Transitional	ns	0.0951
SPClowMHChigh vs. Transitional	ns	0.0634		SPClowMHChigh vs. Transitional	**	0.0041
Day 3				Day 24		
AT2 vs. Multiciliated	****	<0.0001		AT2 vs. Multiciliated	****	<0.0001
AT2 vs. Club	ns	0.4136		AT2 vs. Club	*	0.0217
AT2 vs. SPClowMHChigh	ns	0.8747		AT2 vs. SPClowMHChigh	ns	0.2182
AT2 vs. Transitional	**	0.0019		AT2 vs. Transitional	***	0.0002
Multiciliated vs. Club	****	<0.0001		Multiciliated vs. Club	****	<0.0001
Multiciliated vs. SPClowMHChigh	****	<0.0001		Multiciliated vs. SPClowMHChigh	****	<0.0001
Multiciliated vs. Transitional	****	<0.0001		Multiciliated vs. Transitional	**	0.0043
Club vs. SPClowMHChigh	ns	0.5092		Club vs. SPClowMHChigh	ns	0.2826
Club vs. Transitional	*	0.0209		Club vs. Transitional	ns	0.1366
SPClowMHChigh vs. Transitional	**	0.0031		SPClowMHChigh vs. Transitional	*	0.0108
Day 7				Day 35		
AT2 vs. Multiciliated	****	<0.0001		AT2 vs. Multiciliated	****	<0.0001
AT2 vs. Club	ns	0.0976		AT2 vs. Club	ns	0.4946
AT2 vs. SPClowMHChigh	ns	0.7452		AT2 vs. SPClowMHChigh	ns	0.392
AT2 vs. Transitional	***	0.0006		AT2 vs. Transitional	**	0.0085
Multiciliated vs. Club	****	<0.0001		Multiciliated vs. Club	****	<0.0001
Multiciliated vs. SPClowMHChigh	****	<0.0001		Multiciliated vs. SPClowMHChigh	****	<0.0001
Multiciliated vs. Transitional	****	<0.0001		Multiciliated vs. Transitional	****	<0.0001
Club vs. SPClowMHChigh	ns	0.1823		Club vs. SPClowMHChigh	ns	0.1245
Club vs. Transitional	ns	0.0676		Club vs. Transitional	*	0.0498
SPClowMHChigh vs. Transitional	**	0.0017		SPClowMHChigh vs. Transitional	***	0.0005
Day 8						
AT2 vs. Multiciliated	****	<0.0001				
AT2 vs. Club	****	<0.0001				
AT2 vs. SPClowMHChigh	****	<0.0001				
AT2 vs. Transitional	ns	0.0686				
Multiciliated vs. Club	****	<0.0001				
Multiciliated vs. SPClowMHChigh	****	<0.0001				
Multiciliated vs. Transitional	****	<0.0001				
Club vs. SPClowMHChigh	ns	0.5466				
Club vs. Transitional	**	0.0025				
SPClowMHChigh vs. Transitional	*	0.0148				

Supplementary Table 7: List of antibodies used in this study.			
**ANTIBODIES**	**SOURCE**	**CATALOG #**	**DILUTION**
BUV737 CD45.2 (Clone 104)	BD Biosciences	612778	1:200
APC Cy7 EpCAM (Clone G8.8)	Biolegend	118218	1:200
PerCP Cy5.5 I-A/I-E (MHC-II, Clone M5/114.15.2)	BD Biosciences	562363	1:200
PE CD40 (Clone 3/23)	BD Biosciences	553791	1:100
PE Cy7 PD-L1 (Clone 10F.9G2)	Biolegend	124314	1:100
PE PD-L1 (Clone 10F.9G2)	Biolegend	124308	1:100
BV421 CD24 (Clone M1/69)	BD Biosciences	562563	1:200
PE Cy7 CD24 (Clone M1/69)	BD Biosciences	560536	1:100
APC CD104 (Clone 346-11A)	Biolegend	123612	1:100
SB600 Podoplanin (Clone 8.1.1)	Thermofisher	63-5381-82	1:100
PE Sca1 (Clone D7)	BD Biosciences	553108	1:100
Alexa Fluor 700 I-A/I-E (MHC-II, Clone M5/114.15.2)	Biolegend	107621	1:400
Alexa Fluor 532 CD45 (Clone 30-F11)	Invitrogen	58-0451-82	1:800
BV510 CD54/ICAM1 (Clone 3E2)	BD Biosciences	563628	1:800
PE Dazzle594 CD80 (Clone 16-10A1)	Biolegend	104737	1:200
BV650 CD86 (Clone GL-1)	Biolegend	105035	1:400
BV480 PD-L2 (Clone TY-25)	BD Biosciences	746756	1:200
eFluor450 VCAM1 (clone 429)	Invitrogen	48-1061-80	1:50
Biotin ICOS-L (Clone HK5.3)	Biolegend	107403	1:100
FITC Streptavidin	Biolegend	405202	1:800
BV510 CD45 (Clone HI30)	Biolegend	304036	1:20
FITC EpCAM (Clon 9C4)	Biolegend	324204	1:20
BV421 HLA-DR,DP,DQ (MHC-II, Clone TU39)	BD Biosciences	564244	1:20
PE Cy7 PD-L1 (Clone 29E.2A3)	Biolegend	329718	1:20
BV510 PD-1 (Clone 29F.1A12)	Biolegend	135241	1:200
BV570 CD44 (Clone IM7)	Biolegend	103037	1:50
BV605 CD19 (Clone 6D5)	Biolegend	115539	1:200
BV650 CD62L (Clone MEL-14)	BD Biosciences	564108	1:800
BV786 CD11a (CloneM17/4)	BD Biosciences	740866	1:200
PE CD69 (Clone H1.2F3)	Biolegend	104508	1:100
PE Cy5.5 CD25 (Clone PC61.5)	Invitrogen	35-0251-80	1:200
Alexa Fluor 647 CD3e (Clone 145-2C11)	Biolegend	100322	1:400
Alexa Fluor 700 CD4 (Clone RM4-4)	Biolegend	116021	1:800
Alexa Fluor 488 CD8a (53-6.7)	Biolegend	100723	1:800
BUV395 CD3(Clone 145-2C11)	BD Biosciences	563565	1:50
BUV805 CD8a (Clone 53-6.7)	BD Biosciences	612898	1:80
eFluor450 Ki67 (clone Sola15)	Invitrogen	48-5698-80	1:800
BV711 CD103 (Clone 2E7)	Biolegend	121435	1:100
FITC FOXP3 (Clone FJK-16s)	Invitrogen	11-5773-82	1:100
PE-e610 GATA-3 (Clone TWAJ)	Invitrogen	61-9966-41	1:20
PE Cy7 T-bet (Clone 4B10)	Biolegend	644823	1:100
APC RORgT (Clone B2D)	Invitrogen	17-6981-80	1:100
Alexa Fluor 488 CD45 (Clone 30-F11)	Biolegend	103122	1:100
BV510 CD4 (Clone GK1.5)	Biolegend	100449	1:100
APC CD11a (Clone M17/4)	Invitrogen	17-0111-82	1:100
BV421 CD44 (Clone IM7)	BD Biosciences	563970	1:100
PE Cy7 CD62L (Clone MEL-14)	Biolegend	104418	1:100
APC Cy7 CD8a (Clone 53-6.7)	Biolegend	100714	1:100
PerCP Cy5.5 CD103 (Clone 2E7)	Biolegend	121416	1:100
BV605 CD4 (Clone GK1.5)	Biolegend	100451	1:100
PerCP Cy5.5 CD45 (Clone 30-F11)	Biolegend	103132	1:100
PE Cy7 CD3e (Clone 145-2C11)	Biolegend	100320	1:50
FITC IL-17A (Clone TC11-18H10.1)	Biolegend	506908	1:50
APC IL-5 (Clone TRFK5)	Biolegend	504306	1:20
PE IL-13 (Clone eBio13A)	Invitrogen	12-7133-41	1:20
APC Cy7 IFN-γ (Clone XMG1.2)	Biolegend	505850	1:50
BV421 IL-4 (Clone 11B11)	Biolegend	504120	1:20
PE Cy7 CD11c (Clone HL3)	BD Biosciences	558079	1:100
eFluor450 Ly-6C (Clone HK1.4)	Invitrogen	48-5932-82	1:200
PE CD103 (Clone 2E7)	Biolegend	121406	1:200
BV510 CD45 (Clone 30-F11)	BD Biosciences	563891	1:200
FITC CD64 (Clone X54-5/7.1)	Biolegend	139316	1:200
APC Cy7 Siglec-F (Clone E50-2440)	BD Biosciences	565527	1:200
APC Ly-6G (Clone 1A8)	BD Biosciences	560599	1:200
BUV395 CD11b (Clone M1/70)	BD Biosciences	563553	1:200
Pure anti-mouse CD16/32 Fc Block (Clone 93)	Biolegend	101302	1:20
Human TruStain FcX™ Fc Block	Biolegend	422302	1:20
rabbit anti-mouse CD4	Abcam	ab183685	1:500
InVivoMab anti-mouse CD154 (CD40L) (Clone MR-1)	BioXCell	BE0017-1	See Methods
InVivoMAb polyclonal Armenian hamster IgG control	BioXCell	BE0091	See Methods

Supplementary Table 8: qRTPCR Probe Context Sequences

Target	TaqMan Assay ID #	Probe Context Sequence (5' to 3')
Spc	Mm00488144_m1	GAGTCCACCGGATTACTCGGCAGGT
Scgb1a1	Mm00442046_m1	GCTGCAGCTCAGCTTCTTCGGACAT
Foxj1	Mm01267279_m1	GACCCCACCTGGCAGAATTCCATCC
Aqp5	Mm00437578_m1	TGTGGGGATCTACTTCACCGGCTGT
Aw112010	Mm01197675_m1	ATGTCTCCCATCCCTCTGATATTTA
Sox2	Mm03053810_s1	GCTGCCGAGAATCCATGTATATATT
H2-K1	Mm01612247_mH	GCTGCTGCGCACAGATTCCCCAAAG
Cdkn1a	Mm00432448_m1	GACCAGCCTGACAGATTTCTATCAC
Cd74	Mm00658576_m1	CTAGAGAGCCAGAAAGGTGCAGCCG
H2-Dma	Mm00439226_m1	TCGAAGCATCTACACCAGTGTTCTG
H2-DMb1	Mm04213366_s1	ATAATGAAGTCTGTCCCAGGTGGGG
H2-DMb2	Mm00783707_s1	CAACAAAGATCTGCTGGCCTGCTGG
H2-Oa	Mm00468476_m1	CCATCAAGGCCGACCACATGGGCTC
H2-Ob	Mm00468801_m1	ACTCCCCAGAGAATTTTGTGATTCA
Cd274 (Pd-l1)	Mm03048248_m1	GAGAAAACAAGTGAGAATGCTAGAT
