# Supplementary material for: Antigen presentation by lung epithelial cells directs CD4+ TRM cell function and regulates barrier immunity
Source: Nat Commun. 2021 Oct 5;12:5834. doi: 10.1038/s41467-021-26045-w (PMC8492657; doi:10.1038/s41467-021-26045-w)
Supplement: Supplementary file 3 — Reporting Summary [file 41467_2021_26045_MOESM3_ESM.pdf]

nature portfolio

Corresponding author(s): Joseph P. Mizgerd

Last updated by author(s): Jul 28, 2021

Reporting Summary

Nature Portfolio wishes to improve the reproducibility of the work that we publish. This form provides structure for consistency and transparency in reporting. For further information on Nature Portfolio policies, see our Editorial Policies and the Editorial Policy Checklist.

Statistics

For all statistical analyses, confirm that the following items are present in the figure legend, table legend, main text, or Methods section.

n/a	Confirmed	
☐	☒	The exact sample size (n) for each experimental group/condition, given as a discrete number and unit of measurement
☐	☒	A statement on whether measurements were taken from distinct samples or whether the same sample was measured repeatedly
☐	☒	The statistical test(s) used AND whether they are one- or two-sided<br>*Only common tests should be described solely by name; describe more complex techniques in the Methods section.*
☒	☐	A description of all covariates tested
☐	☒	A description of any assumptions or corrections, such as tests of normality and adjustment for multiple comparisons
☐	☒	A full description of the statistical parameters including central tendency (e.g. means) or other basic estimates (e.g. regression coefficient) AND variation (e.g. standard deviation) or associated estimates of uncertainty (e.g. confidence intervals)
☐	☒	For null hypothesis testing, the test statistic (e.g. F, t, r) with confidence intervals, effect sizes, degrees of freedom and P value noted<br>*Give P values as exact values whenever suitable.*
☒	☐	For Bayesian analysis, information on the choice of priors and Markov chain Monte Carlo settings
☒	☐	For hierarchical and complex designs, identification of the appropriate level for tests and full reporting of outcomes
☐	☒	Estimates of effect sizes (e.g. Cohen's d, Pearson's r), indicating how they were calculated

Our web collection on statistics for biologists contains articles on many of the points above.

Software and code

Policy information about availability of computer code

Data collection	SpectraFlo (Cytek), FACSDiva (BD Biosciences), Leica LAS X were used for collection flow cytometry, multispectral cytometry and immunofluorescence images respectively.
Data analysis	FlowJo software Version 10.8.0 (BD Biosciences) and ImageJ Version 2.0.0.-rc-69 were used to analyze flowcytometry and immunofluorescence data respectively. Omiq.ai cloud computation platform (Omiq) was used to analyze the multispectral cytometry data. Statistical analyses were performed using GraphPad Prism Version 9.1.2 (225)

For manuscripts utilizing custom algorithms or software that are central to the research but not yet described in published literature, software must be made available to editors and reviewers. We strongly encourage code deposition in a community repository (e.g. GitHub). See the Nature Portfolio guidelines for submitting code & software for further information.

Data

Policy information about availability of data

All manuscripts must include a data availability statement. This statement should provide the following information, where applicable:

- Accession codes, unique identifiers, or web links for publicly available datasets
- A description of any restrictions on data availability
- For clinical datasets or third party data, please ensure that the statement adheres to our policy

Original data generated in this study are available within the paper and associated supplementary files, with additional information available from the corresponding author upon reasonable request. Source data are available as a “Source data” file provided with this paper.

Field-specific reporting

Please select the one below that is the best fit for your research. If you are not sure, read the appropriate sections before making your selection.

- [x] Life sciences
- [ ] Behavioural & social sciences
- [ ] Ecological, evolutionary & environmental sciences

For a reference copy of the document with all sections, see nature.com/documents/nr-reporting-summary-flat.pdf

Life sciences study design

All studies must disclose on these points even when the disclosure is negative.

Sample size	No sample size calculation was performed. Sample sizes were determined based on prior studies and experience (Smith et al, Mucosal Immunology 2018; Shenoy et al. Mucosal Immunology 2020; Guillon et al. JCI Insights 2020; Barker et al. JCI 2021.)
Data exclusions	All MHC-IIΔEpi mice without littermate controls were excluded. WT mice among the litters of SPC-GFP mice were excluded due absence of requisite genetic loci. Experimental outliers were identified as mice that were affected by improper surgeries and/or bacterial instillations leading to overt tissue pathologies based on prior studies and experience. Experimental outliers were excluded from in data Figures. 4f,6f,6g. Statistical outliers were identified using the Grubbs Test (alpha=0.05) on GraphPad Prism. Statistical outliers were excluded from data in Figures. 2b, 3i, 4d, 3e, 5f, and Supplementary Figures. 1d.
Replication	The exact number of experimental replicates with the sample size per experiment are designated in the figure legends. All experiments were repeated as 2 independent experiments (also mentioned in the legends).
Randomization	Animals were assigned to experimental and control groups randomly. For microscopy imaging and analyses, 15 images of randomly selected fields per coronal section from each mouse lung were identified in an unbiased raster fashion, from the top left to bottom right of the lung section.
Blinding	For experiments with C57BL6/J mice, mice had to be randomly assigned to separate cages for different infections, timepoints and treatments. Hence, the investigators were not blinded during experimentation. However, during data collection, all mice were assigned a ID to ensure blinding and all analyses on the data was performed in a blinded fashion. For analyses of data involving SPC-GFP and MHC-IIΔEpi mice, the mice were assigned a unique numerical code and all analyses on the data was performed in a blinded fashion. Blinding was also used during imaging and analysis of the electron microscopy and immunofluorescent quantification for temporal CD4 T cell densities in the lungs.

Reporting for specific materials, systems and methods

We require information from authors about some types of materials, experimental systems and methods used in many studies. Here, indicate whether each material, system or method listed is relevant to your study. If you are not sure if a list item applies to your research, read the appropriate section before selecting a response.

Materials & experimental systems

n/a	Involved in the study	
[ ]	[x]	Antibodies
[x]	[ ]	Eukaryotic cell lines
[x]	[ ]	Palaeontology and archaeology
[ ]	[x]	Animals and other organisms
[x]	[ ]	Human research participants
[x]	[ ]	Clinical data
[x]	[ ]	Dual use research of concern

Methods

n/a	Involved in the study	
[x]	[ ]	ChIP-seq
[ ]	[x]	Flow cytometry
[x]	[ ]	MRI-based neuroimaging

Antibodies

Antibodies used	All commercially available antibodies have been validated by the supplier for their target and application, with relevant dilution, publications and validation information available on the manufacturer's websites for each antibody. All this information can be found on the website corresponding to each specific antibody by using the catalog number listed in Supplementary Table 8. The URLs are also below listed with the antibodies in the reporting summary.<br><br>BUV737 CD45.2 (Clone 104) BD Biosciences 612778 https://www.bdbiosciences.com/en-us/products/reagents/flow-cytometry-reagents/research-reagents/single-color-antibodies-ruo/buv737-mouse-anti-mouse-cd45-2.612778<br>APC Cy7 EpCAM (Clone G8.8) Biolegend 118218 https://www.biolegend.com/en-us/products/apc-cyanine7-anti-mouse-cd326-ep-cam-antibody-5577?GroupID=BLG5748<br>PerCP Cy5.5 I-A/I-E (MHC-II, Clone M5/114.15.2) BD Biosciences 562363 https://www.bdbiosciences.com/en-us/products/reagents/flow-cytometry-reagents/research-reagents/single-color-antibodies-ruo/percp-cy-5-5-rat-anti-mouse-i-a-i-e.562363<br>PE CD40 (Clone 3/23) BD Biosciences 553791 https://www.bdbiosciences.com/en-us/products/reagents/flow-cytometry-reagents/research-reagents/single-color-antibodies-ruo/pe-rat-anti-mouse-cd40.553791

PE Cy7 PD-L1 (Clone 10F.9G2) Biolegend 124314 https://www.biolegend.com/en-us/products/pe-cyanine7-anti-mouse-cd274-b7-h1-pd-l1-antibody-6721?GroupID=BLG5396
PE PD-L1 (Clone 10F.9G2) Biolegend 124308 https://www.biolegend.com/en-us/products/pe-anti-mouse-cd274-b7-h1-pd-l1-antibody-4497
BV421 CD24 (Clone M1/69) BD Biosciences 562563 bdbiosciences.com/en-us/products/reagents/flow-cytometry-reagents/research-reagents/single-color-antibodies-ruo/bv421-rat-anti-mouse-cd24.562563
PE Cy7 CD24 (Clone M1/69) BD Biosciences 560536 https://www.bdbiosciences.com/en-tw/products/reagents/flow-cytometry-reagents/research-reagents/single-color-antibodies-ruo/pe-cy-7-rat-anti-mouse-cd24.560536
APC CD104 (Clone 346-11A) Biolegend 123612 https://www.biolegend.com/en-us/products/apc-anti-mouse-cd104-antibody-16031?GroupID=BLG5356
SB600 Podoplanin (Clone 8.1.1) Thermofisher 63-5381-82 https://www.thermofisher.com/antibody/product/Podoplanin-Antibody-clone-eBio8-1-1-8-1-1-Monoclonal/63-5381-82
PE Sca1 (Clone D7) BD Biosciences 553108 https://www.bdbiosciences.com/en-us/products/reagents/flow-cytometry-reagents/research-reagents/single-color-antibodies-ruo/pe-rat-anti-mouse-ly-6a-e.553108
Alexa Fluor 700 I-A/I-E (MHC-II, Clone M5/114.15.2) Biolegend 107621 https://www.biolegend.com/en-us/search-results/alexa-fluor-700-anti-mouse-i-a-i-e-antibody-3413
Alexa Fluor 532 CD45 (Clone 30-F11) Invitrogen 58-0451-82 https://www.thermofisher.com/antibody/product/CD45-Antibody-clone-30-F11-Monoclonal/58-0451-82
BV510 CD54/ICAM1 (Clone 3E2) BD Biosciences 563628 https://www.bdbiosciences.com/en-us/products/reagents/flow-cytometry-reagents/research-reagents/single-color-antibodies-ruo/bv510-hamster-anti-mouse-cd54.563628
PE Dazzle594 CD80 (Clone 16-10A1) Biolegend 104737 https://www.biolegend.com/en-us/search-results/pe-dazzle-594-anti-mouse-cd80-antibody-10221
BV650 CD86 (Clone GL-1) Biolegend 105035 https://www.biolegend.com/en-us/products/brilliant-violet-650-anti-mouse-cd86-antibody-7643?GroupID=BLG10719
BV480 PD-L2 (Clone TY-25) BD Biosciences 746756 https://www.bdbiosciences.com/en-us/products/reagents/flow-cytometry-reagents/research-reagents/single-color-antibodies-ruo/bv480-rat-anti-mouse-cd273.746756
eFluor450 VCAM1 (clone 429) Invitrogen 48-1061-80 https://www.thermofisher.com/antibody/product/CD106-VCAM-1-Antibody-clone-429-Monoclonal/48-1061-82
Biotin ICOS-L (Clone HK5.3) Biolegend 107403 https://www.biolegend.com/en-us/products/biotin-anti-mouse-cd275-b7-h2-b7-rp1-icos-ligand-antibody-2550?GroupID=BLG3777
FITC Streptavidin Biolegend 405202 https://www.biolegend.com/en-us/products/fitc-streptavidin-1473?GroupID=GROUP23
BV510 CD45 (Clone HI30) Biolegend 304036 https://www.biolegend.com/en-us/products/brilliant-violet-510-anti-human-cd45-antibody-8006
FITC EpCAM (Clon 9C4) Biolegend 324204 https://www.biolegend.com/en-us/products/fitc-anti-human-cd326-epcam-antibody-3756
BV421 HLA-DR,DP,DQ (MHC-II, Clone TU39) BD Biosciences 564244 https://www.bdbiosciences.com/en-us/products/reagents/flow-cytometry-reagents/research-reagents/single-color-antibodies-ruo/bv421-mouse-anti-human-hla-dr-dp-dq.564244
PE Cy7 PD-L1 (Clone 29E.2A3) Biolegend 329718 https://www.biolegend.com/en-us/products/pe-cyanine7-anti-human-cd274-b7-h1-pd-l1-antibody-8277?GroupID=BLG5402
BV510 PD-1 (Clone 29F.1A12) Biolegend 135241 https://www.biolegend.com/en-us/search-results/brilliant-violet-510-anti-mouse-cd279-pd-1-antibody-14923?GroupID=BLG7928
BV570 CD44 (Clone IM7) Biolegend 103037 https://www.biolegend.com/en-us/search-results/brilliant-violet-570-anti-mouse-human-cd44-antibody-7386
BV605 CD19 (Clone 6D5) Biolegend 115539 https://www.biolegend.com/en-us/products/brilliant-violet-605-anti-mouse-cd19-antibody-7645?GroupID=BLG10556
BV650 CD62L (Clone MEL-14) BD Biosciences 564108 https://www.bdbiosciences.com/en-us/products/reagents/flow-cytometry-reagents/research-reagents/single-color-antibodies-ruo/bv650-rat-anti-mouse-cd62l.564108
BV786 CD11a (CloneM17/4) BD Biosciences 740866 https://www.bdbiosciences.com/en-us/products/reagents/flow-cytometry-reagents/research-reagents/single-color-antibodies-ruo/bv786-rat-anti-mouse-cd11a.740866
PE CD69 (Clone H1.2F3) Biolegend 104508 https://www.biolegend.com/en-us/products/pe-anti-mouse-cd69-antibody-265?GroupID=BLG10515
PE Cy5.5 CD25 (Clone PC61.5) Invitrogen 35-0251-80 https://www.thermofisher.com/antibody/product/CD25-Antibody-clone-PC61-5-Monoclonal/35-0251-82
Alexa Fluor 647 CD3e (Clone 145-2C11) Biolegend 100322 https://www.biolegend.com/en-us/search-results/alexa-fluor-647-anti-mouse-cd3epsilon-antibody-2677
Alexa Fluor 700 CD4 (Clone RM4-4) Biolegend 116021 https://www.biolegend.com/en-us/search-results/alexa-fluor-700-anti-mouse-cd4-antibody-14801?GroupID=BLG4211
Alexa Fluor 488 CD8a (53-6.7) Biolegend 100723 https://www.biolegend.com/en-us/products/alexa-fluor-488-anti-mouse-cd8a-antibody-2698?GroupID=BLG6765
BUV395 CD3(Clone 145-2C11) BD Biosciences 563565 https://www.bdbiosciences.com/en-us/products/reagents/flow-cytometry-reagents/research-reagents/single-color-antibodies-ruo/buv395-hamster-anti-mouse-cd3e.563565
BUV805 CD8a (Clone 53-6.7) BD Biosciences 612898 https://www.bdbiosciences.com/en-us/products/reagents/flow-cytometry-reagents/research-reagents/single-color-antibodies-ruo/buv805-rat-anti-mouse-cd8a.612898
eFluor450 Ki67 (clone Sola15) Invitrogen 48-5698-80 https://www.thermofisher.com/antibody/product/Ki-67-Antibody-clone-SolA15-Monoclonal/48-5698-82
BV711 CD103 (Clone 2E7) Biolegend 121435 https://www.biolegend.com/de-at/products/brilliant-violet-711-anti-mouse-cd103-antibody-14411?GroupID=BLG4646
FITC FOXP3 (Clone FJK-16s) Invitrogen 11-5773-82 https://www.thermofisher.com/antibody/product/FOXP3-Antibody-clone-FJK-16s-Monoclonal/11-5773-82
PE-e610 GATA-3 (Clone TWAJ) Invitrogen 61-9966-41 https://www.thermofisher.com/antibody/product/Gata-3-Antibody-clone-TWAJ-Monoclonal/61-9966-42
PE Cy7 T-bet (Clone 4B10) Biolegend 644823 https://www.biolegend.com/en-us/products/pe-cyanine7-anti-t-bet-antibody-8328?GroupID=BLG6433

APC RORgT (Clone B2D) Invitrogen 17-6981-80 https://www.thermofisher.com/antibody/product/17-6981-82.html?CID=AFLLS-17-6981-82
Alexa Fluor 488 CD45 (Clone 30-F11) Biolegend 103122 https://www.biolegend.com/en-us/search-results/alexa-fluor-488-anti-mouse-cd45-antibody-3100
BV510 CD4 (Clone GK1.5) Biolegend 100449 https://www.biolegend.com/en-us/products/brilliant-violet-510-anti-mouse-cd4-antibody-10707?GroupID=BLG4745
APC CD11a (Clone M17/4) Invitrogen 17-0111-82 https://www.thermofisher.com/antibody/product/CD11a-LFA-1alpha-Antibody-clone-M17-4-Monoclonal/17-0111-82
BV421 CD44 (Clone IM7) BD Biosciences 563970 https://www.bdbiosciences.com/en-us/products/reagents/flow-cytometry-reagents/research-reagents/single-color-antibodies-ruo/bv421-rat-anti-mouse-cd44.563970
PE Cy7 CD62L (Clone MEL-14) Biolegend 104418 https://www.biolegend.com/en-us/products/pe-cyanine7-anti-mouse-cd62l-antibody-1922?GroupID=BLG10670
APC Cy7 CD8a (Clone 53-6.7) Biolegend 100714 https://www.biolegend.com/en-us/search-results/apc-cyanine7-anti-mouse-cd8a-antibody-2269?GroupID=BLG279
PerCP Cy5.5 CD103 (Clone 2E7) Biolegend 121416 https://www.biolegend.com/en-us/products/percp-cyanine5-5-anti-mouse-cd103-antibody-5599?GroupID=BLG4646
BV605 CD4 (Clone GK1.5) Biolegend 100451 https://www.biolegend.com/en-us/products/brilliant-violet-605-anti-mouse-cd4-antibody-10708?GroupID=BLG4745
PerCP Cy5.5 CD45 (Clone 30-F11) Biolegend 103132 https://www.biolegend.com/en-us/products/percp-cyanine5-5-anti-mouse-cd45-antibody-4264?GroupID=BLG6829
PE Cy7 CD3e (Clone 145-2C11) Biolegend 100320 https://www.biolegend.com/en-us/products/pe-cyanine7-anti-mouse-cd3epsilon-antibody-1899
FITC IL-17A (Clone TC11-18H10.1) Biolegend 506908 https://www.biolegend.com/de-at/products/fitc-anti-mouse-il-17a-antibody-3534
APC IL-5 (Clone TRFK5) Biolegend 504306 https://www.biolegend.com/en-us/search-results/apc-anti-mouse-human-il-5-antibody-989
PE IL-13 (Clone eBio13A) Invitrogen 12-7133-41 https://www.thermofisher.com/antibody/product/IL-13-Antibody-clone-eBio13A-Monoclonal/12-7133-41
APC Cy7 IFN- (Clone XMG1.2) Biolegend 505850 https://www.biolegend.com/en-us/sean-tuckers-tests/apc-cyanine7-anti-mouse-ifn-gamma-antibody-13155?GroupID=GROUP24
BV421 IL-4 (Clone 11B11) Biolegend 504120 https://www.biolegend.com/en-us/search-results/brilliant-violet-421-anti-mouse-il-4-antibody-7306
PE Cy7 CD11c (Clone HL3) BD Biosciences 558079 https://www.bdbiosciences.com/en-us/products/reagents/flow-cytometry-reagents/research-reagents/single-color-antibodies-ruo/pe-cy-7-hamster-anti-mouse-cd11c.561022
eFluor450 Ly-6C (Clone HK1.4) Invitrogen 48-5932-82 https://www.thermofisher.com/antibody/product/Ly-6C-Antibody-clone-HK1-4-Monoclonal/48-5932-82
PE CD103 (Clone 2E7) Biolegend 121406 https://www.biolegend.com/en-us/search-results/pe-anti-mouse-cd103-antibody-3574
BV510 CD45 (Clone 30-F11) BD Biosciences 563891 https://www.bdbiosciences.com/en-us/products/reagents/flow-cytometry-reagents/research-reagents/single-color-antibodies-ruo/bv510-rat-anti-mouse-cd45.563891
FITC CD64 (Clone X54-5/7.1) Biolegend 139316 https://www.biolegend.com/en-us/search-results/fitc-anti-mouse-cd64-fcgammari-antibody-12422?GroupID=BLG8805
APC Cy7 Siglec-F (Clone E50-2440) BD Biosciences 565527 https://www.bdbiosciences.com/en-us/products/reagents/flow-cytometry-reagents/research-reagents/single-color-antibodies-ruo/apc-cy-7-rat-anti-mouse-siglec-f.565527
APC Ly-6G (Clone 1A8) BD Biosciences 560599 https://www.bdbiosciences.com/en-us/products/reagents/flow-cytometry-reagents/research-reagents/single-color-antibodies-ruo/apc-rat-anti-mouse-ly-6g.560599
BUV395 CD11b (Clone M1/70) BD Biosciences 563553 https://www.bdbiosciences.com/en-us/products/reagents/flow-cytometry-reagents/research-reagents/single-color-antibodies-ruo/buv395-rat-anti-cd11b.565976
Pure anti-mouse CD16/32 Fc Block (Clone 93) Biolegend 101302 https://www.biolegend.com/en-us/search-results/purified-anti-mouse-cd16-32-antibody-190
Human TruStain FcX™ Fc Block Biolegend 422302 https://www.biolegend.com/en-us/products/human-trustain-fcx-fc-receptor-blocking-solution-6462?GroupID=BLG2181
rabbit anti-mouse CD4 Abcam ab183685 https://www.abcam.com/cd4-antibody-epr19514-ab183685.html
InVivoMab anti-mouse CD154 (CD40L) (Clone MR-1) BioXCell BE0017-1 https://bxcell.com/product/m-cd154-cd40l/
InVivoMAb polyclonal Armenian hamster IgG control BioXCell BE0091 https://bxcell.com/product/polyclonal-3/

Validation

All commercially available antibodies have been validated by the supplier for the indicated target and application, with relevant dilution, publications and validation information available on the manufacturer's websites for each antibody. All this information can be found on the website corresponding to each specific antibody by using the catalog number listed in Supplementary Table 7. The URLs are also listed with the antibodies in the reporting summary.
In all flow cytometry and IF experiments, positive controls were used to confirm antibody specificity. Negative and fluorescence minus one (FMO) controls were used to account for non-specific staining. For IF, primary Antibody only and fluorophore- tagged secondary Antibody only controls were used to account for any nonspecific staining (see methods for further details).

Animals and other organisms

Policy information about studies involving animals; ARRIVE guidelines recommended for reporting animal research

Laboratory animals

C57BL/6J (Stock# 000664), OT-II TCR transgenic (B6.Cg-Tg(TcraTcrb)425Cbn/J) (Stock # 004194), and B6.Cg-Pdcd1tm1.1Shr/J (PD-1-/-, Stock #028276)42 mice were obtained from The Jackson laboratories (USA) at 6 weeks old age, all on the C57BL/6 background. SPC-GFP mice20 were back-crossed more than 15 generations onto C57BL/6 and bred in Boston University animal facilities. Mice for targeted deletion of MHC-II on LECs were generated in-house at BU-ABSL2 facility. Briefly, male B6.129X1-H2-Ab1tm1Koni/J (Stock# 013181; C57BL/6 background) and female Nkx2-1tm1.1(cre/ERT2)Zjh/J mice (Stock# 014552; B6.129SF2 background) were obtained

from The Jackson Laboratories (USA) and crossed to create Nkx2.1creERT2H2-Ab1fl/fl mice. Both founder mice lack I-E due to mutation in H2-Ea locus allowing deletion of H2-Ab1 to abrogate MHC-II entirely. Nkx2-1 is expressed in all LECs and some cells of telencephalon, thyroid and pituitary67, 68, 69 and Nkx2-1-driven Cre efficiently targets all LECs70. All breeders were homozygous floxed for H2-Ab1. For experiments all mice were homozygous for loxP sites flanking H2-Ab1 exon1 and were identified as Cre-positive or negative based on presence or absence of Nkx2.1cre/ERT2. Cage and littermate controls of both sexes were used for the studies. BASC Reporter mice including the BASC viewer and the BASC v-race mice were on a mixed hybrid C57BL/6 x 129/SV background21. All mice were housed in specific pathogen free environment on a 12-hour light-dark cycle at 30-70%, humidity and temperature of 20-26°C, with ad libitum access to standard chow and water. 7-14-week-old mice were used for all experiments. Mice were euthanized using isoflurane overdose and death confirmed using pneumothorax before organ collections. Animal procedures were performed with compliance to all relevant ethical regulations for animal testing and research, in the United States in accordance with the Guide for the Care and Use of Laboratory Animals published by the National Institutes of Health (after review and approval by the Institutional Animal Care and Use Committee of Boston University) and in Germany according to the German animal protection law (after review and approval by the Regierungspräsidium Darmstadt, Veterinärdezernat of Hesse, Germany).

Wild animals

No wild animals were used in the study.

Field-collected samples

No field collected samples were used in the study.

Ethics oversight

Animal procedures were performed with compliance to all relevant ethical regulations for animal testing and research, in the United States in accordance with the Guide for the Care and Use of Laboratory Animals published by the National Institutes of Health (after review and approval by the Institutional Animal Care and Use Committee of Boston University) and in Germany according to the German animal protection law (after review and approval by the Regierungspräsidium Darmstadt, Veterinärdezernat of Hesse, Germany).

Note that full information on the approval of the study protocol must also be provided in the manuscript.

Flow Cytometry

Plots

Confirm that:

- [x] The axis labels state the marker and fluorochrome used (e.g. CD4-FITC).
- [x] The axis scales are clearly visible. Include numbers along axes only for bottom left plot of group (a 'group' is an analysis of identical markers).
- [x] All plots are contour plots with outliers or pseudocolor plots.
- [x] A numerical value for number of cells or percentage (with statistics) is provided.

Methodology

Sample preparation

Murine lung digestion for epithelial cell flow cytometry and FACS sorting
For high throughput flow cytometry of lung epithelial cells, elastase digestion of murine lung was performed. Briefly, euthanized mice were exsanguinated, trachea cannulated and lungs lavaged thrice with 1mL Ca-Mg free DPBS containing 0.5mM EDTA solution. The lungs were then inflated with 1mL digestion media (RPMI 1640 containing 10% Dextran, 4.5 units/mL of Elastase (Worthington Biochemicals, Lakewood, NJ) and 150μg/mL of DNase I) and plugged with 0.5mL of 1% low melting temperature agarose in PBS. The heart lung blocks were then placed on petri dishes and covered with ice to allow agarose to solidify for at least 5 minutes. Left lobes of the enzyme-instilled and plugged lungs were dissected and kept for enzymatic digestion in additional 2mL of digestion media for 1 hour at 37°C at 200rpm. The digested lungs were then minced finely using disposable razor blades, washed down with 1mL digestion media and transferred to 50mL conical tubes. The slurry was then kept on shaker for 20mins at 37°C at 200rpm before adding 10mL of RPMI 1640 with 50% heat-inactivated FBS and 100μL of 10mg/mL DNase I. This suspension was then gently vortexed to allow cell aggregate dissociation and incubated on ice for 5 mins. 10mL RPMI 1640 was then added and the suspension shaken vigorously at 300rpm at 37°C before sequential gravity-mediated filtering through 100μm and 70μm cell strainers. The single cell suspensions were then centrifuged at 300g for 10mins to pellet cells before RBC lysis and resuspension in FACS buffer for flow cytometry as per usual procedures. Cells were blocked with TruStain αCD16/CD32 Fc-Block (BioLegend). Flow cytometry was performed on LSR II Flow Cytometer (BD Biosciences). High-dimensional multi-parameter spectral flow cytometry was performed on Aurora (Cytek), SpectraFlo (Cytek) software was utilized for spectral unmixing of the data using ordinary least square algorithm and data were analyzed with FlowJo software (BD Biosciences). Gating strategies are provided in the Supplemental figures and were based on use of Fluorescence minus one (FMO) controls.
For data generated using the BASC reporter mice, dispase digestion was used 21. Briefly, lungs of euthanized mice were perfused with PBS, cannulated via trachea and instilled with 1 ml pre-warmed dispase solution (Corning). The trachea was ligated, the lungs extracted from the thorax and then finely minced using scissors. Two mL dispase and DNase (20 U/ml, Roche) were added to the resulting slurry, and samples digested at 37°C shaker for 20-30 min. Resulting cell suspensions were gentle mixed and sequentially filtered through 100- and 40-μm strainers followed by wash with excess DMEM through the filter. The single cell suspensions were centrifuged (5 min, 300 g), pellets resuspended in 1 mL pre-cooled FACS buffer. Cell suspensions were re-suspended in MACS buffer, mixed with anti-CD45, anti-CD31 and anti-Ter119 microbeads (Miltenyi Biotec), and incubated at 4°C for 15 min. After washing, cells were loaded onto pre-conditioned MS columns that had been placed in the magnetic field of a MACS separator and the flow-through containing unlabeled cells was collected. These enriched LECs were then blocked using CD16/CD32 Fc-Block (BD Biosciences), stained and washed before flow cytometry was performed on LSRFortessa Flow Cytometer (BD Biosciences). Gating was based on use of Fluorescence minus one (FMO) controls. List of antibodies used in this study are provided in Supplementary Table 7.
For RNA-profiling, epithelial subsets from stained single cell suspensions isolated from elastase digested lungs were sorted into RPMI 1640 with 20% FBS on ice using FACS-Aria II SORP (BD Biosciences) before proceeding to RNA extraction.

For TEM analyses, stained LEC cell suspensions were fixed using 2% paraformaldehyde in PBS before sorting using the MoFlo Astrios cell sorter (Beckman Coulter).

Murine lung digestion for leukocyte flow cytometry
To allow enumeration of extravascular versus intravascular fraction of lung CD4+ T cells, anesthetized mice were retro-orbitally administered 2µg anti-CD45.2 antibody 3 minutes prior to euthanasia. Lungs were collected in RPMI 1640 with 10% FBS before processing for flow cytometry. Single-cell suspensions were prepared by digestion of lungs in type 2 collagenase (Worthington Biochemicals, Lakewood, NJ) and DNase I 19. Cells were blocked with TruStain αCD16/CD32 Fc-Block (BioLegend). Flow cytometry was performed on LSR II Flow Cytometer (BD Biosciences). High-dimensional multi-parameter spectral flow cytometry was performed on Aurora (Cytek), SpectraFlo (Cytek) software was utilized for spectral unmixing of the data using ordinary least square algorithm and and data were analyzed with FlowJo software (BD Biosciences). Gating strategies are provided in the Supplemental figures and were based on use of Fluorescence minus one (FMO) controls. List of antibodies used in this study are provided in Supplementary Table 7.

Lung processing for human lung flow cytometry.
Pathologically deemed normal segments of lung biopsies obtained from tumors resected from patients were collected in RPMI 1640. The lung pieces were minced in 2mL of Digestion media (RPMI containing 10% Dextran, 10units/mL of elastase and 150µg/mL of DNase I) with a disposable razor blade to fine slurry. The slurry was then washed down with 3mL more digestion media and transferred to 50ml conical tubes. The dish was washed with additional 5mL digestion media and transferred to the 50mL tube to have total of 10mL cell slurry. The suspension was shaken at 250rpm for 1 hour at 37°C with intermittent vortexing every 20mins after which 10 mL of RPMI 1640 with 50% heat-inactivated FBS and 200uL of 10mg/mL DNAse I was added. The suspension was vortexed to dissociate aggregates and kept on ice for 5 mins. Additional 10mL RPMI 1640 media was added to bring the final liquid volume to 30mL and the tube shaken at 300 rpm for 10 min at 37°C. The lung minces were sequentially filtered through 100µm and 70µm cell strainers using gravity (no scraping). The single cell suspensions then centrifuged at 300g for 10mins to pellet cells before RBC lysis and resuspension in FACS buffer for flow cytometry as per usual procedures. Cells were blocked with Human TruStain FcXTM- Block (BioLegend). Flow cytometry was performed on LSR II Flow Cytometer (BD Biosciences) and data were analyzed with FlowJo software (BD Biosciences). Gating strategies are provided in the Supplemental figures and are based on use of Fluorescence minus one (FMO) controls. List of antibodies used in this study are provided in Supplementary Table 7.

Instrument

Flow cytometry was performed on LSR II Flow Cytometer (BD Biosciences) or LSRFortessa Flow Cytometer (BD Biosciences). High-dimensional multi-parameter spectral flow cytometry was performed on Aurora (Cytek).

Software

SpectraFlo (Cytek) software was utilized for spectral unmixing of the data using ordinary least square algorithm and data were analyzed with FlowJo software Version 10.8.0 (BD Biosciences).

Cell population abundance

Abundance of cell populations where relevant have been provided in Figures.

Gating strategy

Gating strategies are provided in the Supplemental figures and were based on use of Fluorescence minus one (FMO) controls.

☒ Tick this box to confirm that a figure exemplifying the gating strategy is provided in the Supplementary Information.
